# Created Ex Nihilo: A Near‐Infrared Afterglow Luminescent Framework of Imidazole Derivatives for Bioimaging

**DOI:** 10.1002/anie.9929915

**Published:** 2026-09-02

**Authors:** Zihan Wu, Liping Zhang, Ziwei Wang, Yachao Wang, Gelin Xu, Feng Chi, Dongxia Zhu, Martin R. Bryce, Ben Zhong Tang, Lijie Ren

**Affiliations:** ^1^ Key Laboratory of Nanobiosensing and Nanobioanalysis at Universities of Jilin Province Department of Chemistry Northeast Normal University Changchun Jilin China; ^2^ Department of Neurology, Inst. Translat. Med. The First Affiliated Hospital of Shenzhen University Shenzhen Second People's Hospital Shenzhen China; ^3^ Department of Chemistry Durham University Durham UK; ^4^ School of Science and Engineering Shenzhen Institute of Aggregate Science and Technology The Chinese University of Hong Kong Shenzhen Guangdong China

**Keywords:** aggregation‐induced emission, imidazole, iridium(III) complex, near‐infrared afterglow luminescence, sonodynamic therapy, thrombolysis

## Abstract

Afterglow luminescence probes are limited by (i) a lack of new afterglow scaffolds; (ii) a dependence on initial external light activation, and (iii) aggregation‐caused quenching. Herein, we demonstrate for the first time that Ir(III) complexes of general formula Ir(C^N)_2_(N^N)^+^ PF_6_^−^ with an imidazole‐based N^N ligand exhibit bright afterglow luminescence in the presence of reactive oxygen and nitrogen species (RONS) or under ultrasound (US) irradiation. A series of Ir(III) complexes (Ir‐1–Ir‐4) establish a new afterglow luminescence mechanism based on experimental data and theoretical calculations. The key feature of the present mechanism is the reaction of the ─N═C─N─H unit of the imidazole ligands with various RONS. After modification of the luminescent framework by quinolyl‐triphenylamine C^N ligands, Ir‐4 is endowed with aggregation induced emission (AIE) properties and with enhanced near‐infrared afterglow luminescence. When assembled into water‐soluble nanoparticles (NPs) with the aid of an amphiphilic polymer, Ir‐4@R NPs demonstrated excellent afterglow luminescence imaging and effective sonodynamic therapy in vitro and in vivo. These Ir(III) complexes represent the first examples of an afterglow luminescent molecular framework based on readily available and versatile imidazole ligands, breaking through the existing limitation of molecular structures with afterglow luminescence.

## Graphical abstract

A series of Ir(III) complexes (**Ir‐1**‐**Ir‐4**) based on imidazole derivatives provide a new afterglow luminescence mechanism. The work expands the range of afterglow luminescence frameworks to overcome: (i) a lack of new afterglow scaffolds; (ii) the dependence on initial external light activation; and (iii) aggregation‐caused quenching.

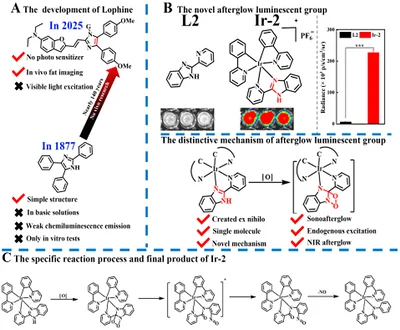

## 1 Introduction

Optical imaging can visualize pathological and physiological processes at the molecular level and is an indispensable tool for preclinical research and biomedical applications [1, 2, 3, 4]. However, traditional fluorescence imaging is hindered by the tissue's own fluorescence, which reduces the imaging sensitivity, specificity, and penetration depth [5, 6, 7, 8]. Afterglow luminescence is a real‐time imaging method which offers unparalleled long‐lasting luminescence with enhanced depth of penetration and spatiotemporal control by avoiding spontaneous fluorescence, light scattering and photobleaching [6, 9, 10, 11, 12, 13, 14, 15, 16, 17]. Although afterglow imaging significantly reduces autofluorescence during the signal acquisition process, it still requires an initial external light activation, which has only shallow tissue penetration and thus hinders the effective activation of afterglow in deep tissues [18]. Unlike light excitation, ultrasound (US) offers deeper tissue penetration (up to more than 4 cm), making it suitable for non‐invasive activation in deep tissues [19, 20, 21]. Activatable afterglow luminescence probes generate afterglow signals in response to disease‐related biomarkers, which further optimizes this method [22, 23]. Unfortunately, many afterglow luminescence studies have been severely limited in their applications by issues of complexity and reproducibility resulting from the doping of multicomponents [2, 24, 25]. This protocol still requires a mixture of photosensitizers, with short afterglow emission wavelengths and shallow tissue penetration. The simple chemical coupling of active components in a single molecule with high‐brightness, near‐infrared (NIR) emission is highly desirable, but its development is still in the infancy stage [17]. Therefore, the development of single molecule probes with sonoafterglow luminescence or activatable afterglow luminescence still faces a serious challenge that requires novel molecular scaffolds.

To date, several self‐sustaining afterglow molecules such as chlorin e4 [2], boron dipyrromethene derivatives (BDI) [26], 3,7‐bis(dibutylamino)‐phenothiazin‐5‐ium salts (mMB) [27], and tetraphenylporphyrin derivatives [28] have been developed via strategies such as structural screening or molecular fusion. The latter utilizes chemical conjugation of a photosensitizer with an afterglow substrate, which is a facile design approach to achieve highly efficient afterglow [29]. However, this strategy confronts the inherent challenge that most self‐sustaining afterglow molecules are incorporated into nanoparticles that often encounter undesirable intermolecular interactions, such as π−π stacking, which can inhibit the photostimulated emission and induce aggregation‐caused quenching (ACQ) of the emission [30]. This effect inevitably hinders the generation of reactive oxygen species (ROS) and significantly reduces the fluorescence quantum yield, thereby severely reducing the signal output [31]. In recent years, organic fluorescent precursors with aggregation‐induced emission properties (AIEgens) have become popular materials for the fabrication of NPs. AIEgens show almost no fluorescence in solution, but when aggregates form, the fluorescence is enhanced due to the restricted internal molecular movement [32, 33], making them more favorable for bioimaging [34]. Therefore, NPs based on AIEgens can provide extremely bright fluorescence and afterglow luminescence. Consequently, constructing ideal afterglow luminescent probes for biological applications requires: (1) a single molecule structure; (2) sonoafterglow or endogenous biomarker stimulation; (3) AIE properties. Nevertheless, to date very few of these afterglow luminescence probes have been explored and used in biomedicine [35].

The auto‐oxidation reaction of the imidazole derivative lophine (2,4,5‐triphenylimidazole) is among the earliest described chemiluminescent reactions [36, 37] first reported by Radziszewski in 1877 [38]. Lophine emits yellow chemiluminescence when reacted with oxygen in the presence of strong alkali [38]. However, over the past 140 years, the application of lophine compounds for chemiluminescence in vivo has received little attention, likely due to the relatively weak chemiluminescence not being suitable for physiological conditions. Recently, Yang et al. observed that caged lophines are capable of strongly emitting near‐infrared light via an afterglow mechanism [39]. Such switching from chemiluminescence to molecular afterglow could overcome the problem of weak intensity for in vivo applications. Despite significant progress in the performance and application of these probes, they are still excited by a blue light‐emitted‐device which can lead to oxidative damage to key biomolecules, including proteins, DNA, and lipids [40]. Transition metal complexes, especially those based on second and third row d^6^ metals such as ruthenium(II) and iridium(III), are renowned for their excellent photophysical properties [41, 42, 43, 44]. The properties of the latter, especially heteroleptic cyclometalated Ir(III) complexes, can be effectively tuned through rational ligand design [45, 46, 47]. Based on the aforementioned research on imidazole derivatives, we introduced benzimidazolyl‐pyridine as the N^N ligands to construct a series of cationic Ir(III) complexes with the structures shown in Figures 1, 2, 3. We unexpectedly discovered that the Ir(III) complexes exhibit strong afterglow luminescence under the action of reactive oxygen and nitrogen species (RONS) or US irradiation (Figure 1) while the free benzimidazolyl‐pyridine ligand did not show any luminescence. This observation establishes two important facts: (i) that the underlying afterglow luminescence mechanism of imidazole compounds is not limited to the oxidation of N–H under alkaline conditions, or the oxidation of ─C═C─ bonds to form a high‐energy intermediate 1,2‐dioxetane [48, 49]; (ii) that the construction of Ir(III) complexes is the key factor that enables the “ex nihilo” turn‐on afterglow luminescence. We propose that the ─N═C─N─H site in the complex can be oxidized by RONS, thereby generating high‐energy intermediates. To our knowledge, this is the first observation of an afterglow luminescent molecular framework based on a cheap and readily available benzimidazolyl‐pyridine ligand.

**FIGURE 1:**
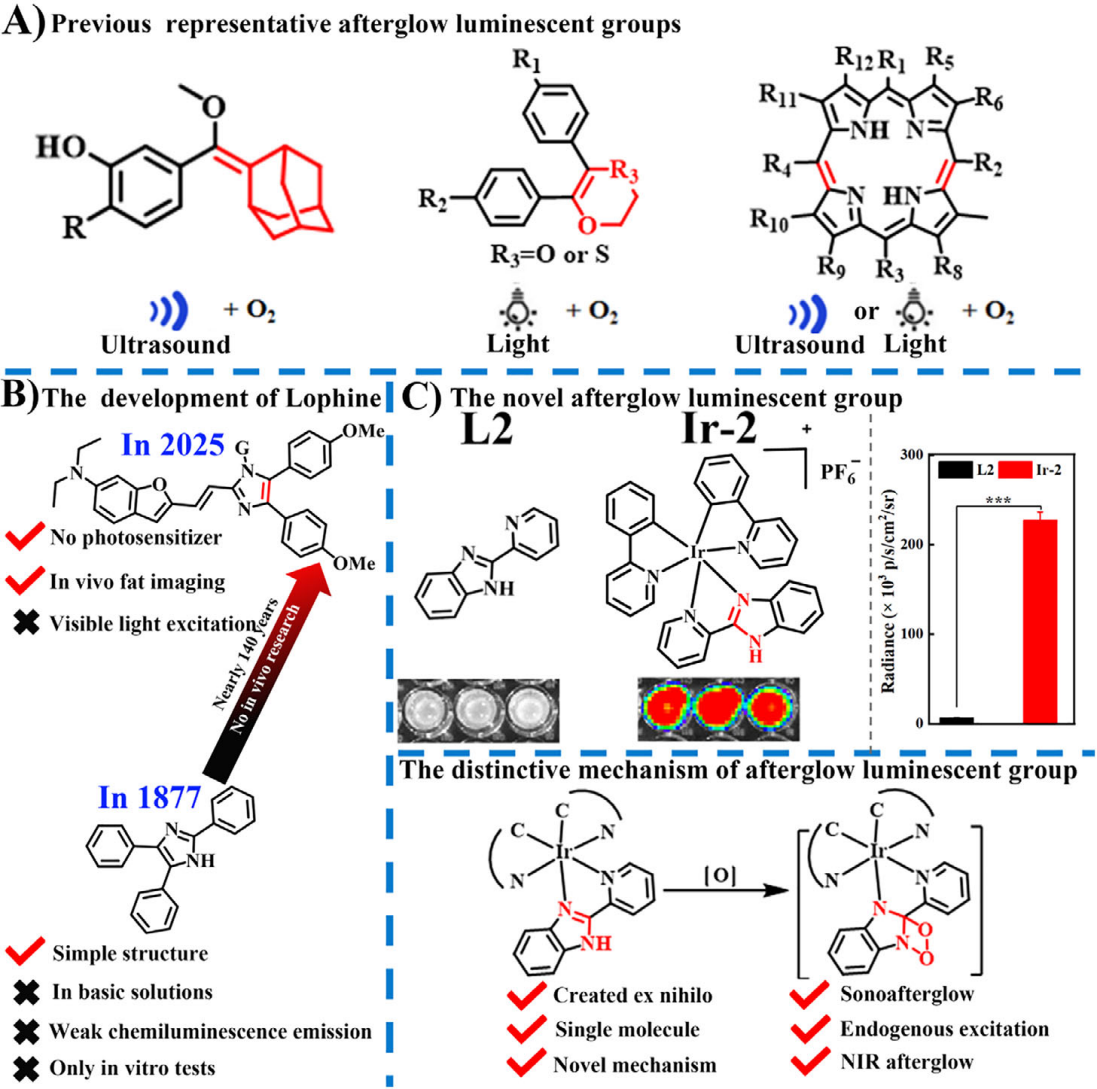
(A) Representative categories of molecules that produce light for afterglow imaging via photo‐initiated [2 + 2] cycloaddition. (B) The development of Lophine. (C) The unique afterglow luminescence mechanism in this work (*Note*: [O] stands for oxidants, ONOO^−^ in this work; the key reactive structural units are shown in red).

**FIGURE 2:**
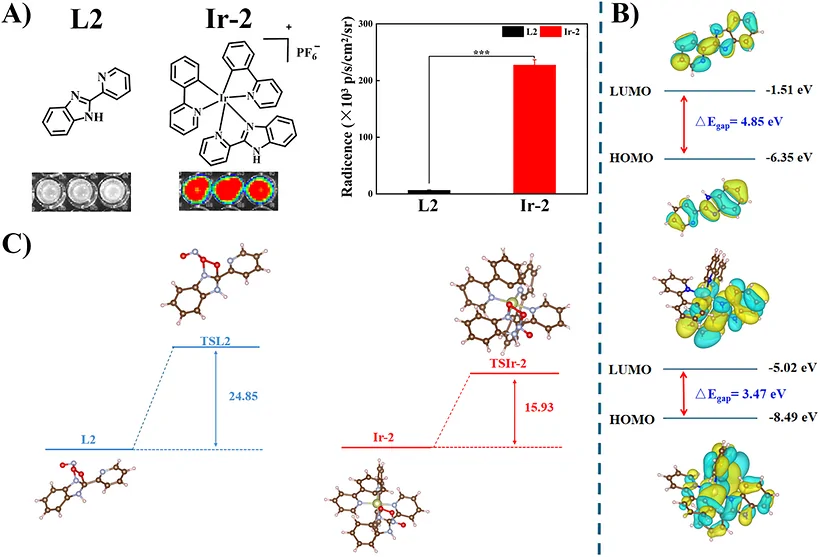
(A) The structural formulas of L2 and Ir‐2. The quantification of corresponding afterglow luminescence intensities in (A). Inset: the afterglow luminescence images acquired by an IVIS imaging system under bioluminescence mode with an open filter. (B) Frontier orbital energies of L2 and Ir‐2 by DFT calculations. (C) Gibbs energy changes (in kcal/mol) of the rate‐determining step in the formation of unstable dioxetane intermediates by ONOO^−^ oxidizing L2 and Ir‐2 (TS is transition state). Data are represented as mean ± SD (*n* = 3). The data were analyzed by unpaired 2‐tailed Student's *t*‐test using the GraphPad Prism 7. *p < 0.05, **p < 0.01, ***p < 0.001.

**FIGURE 3:**
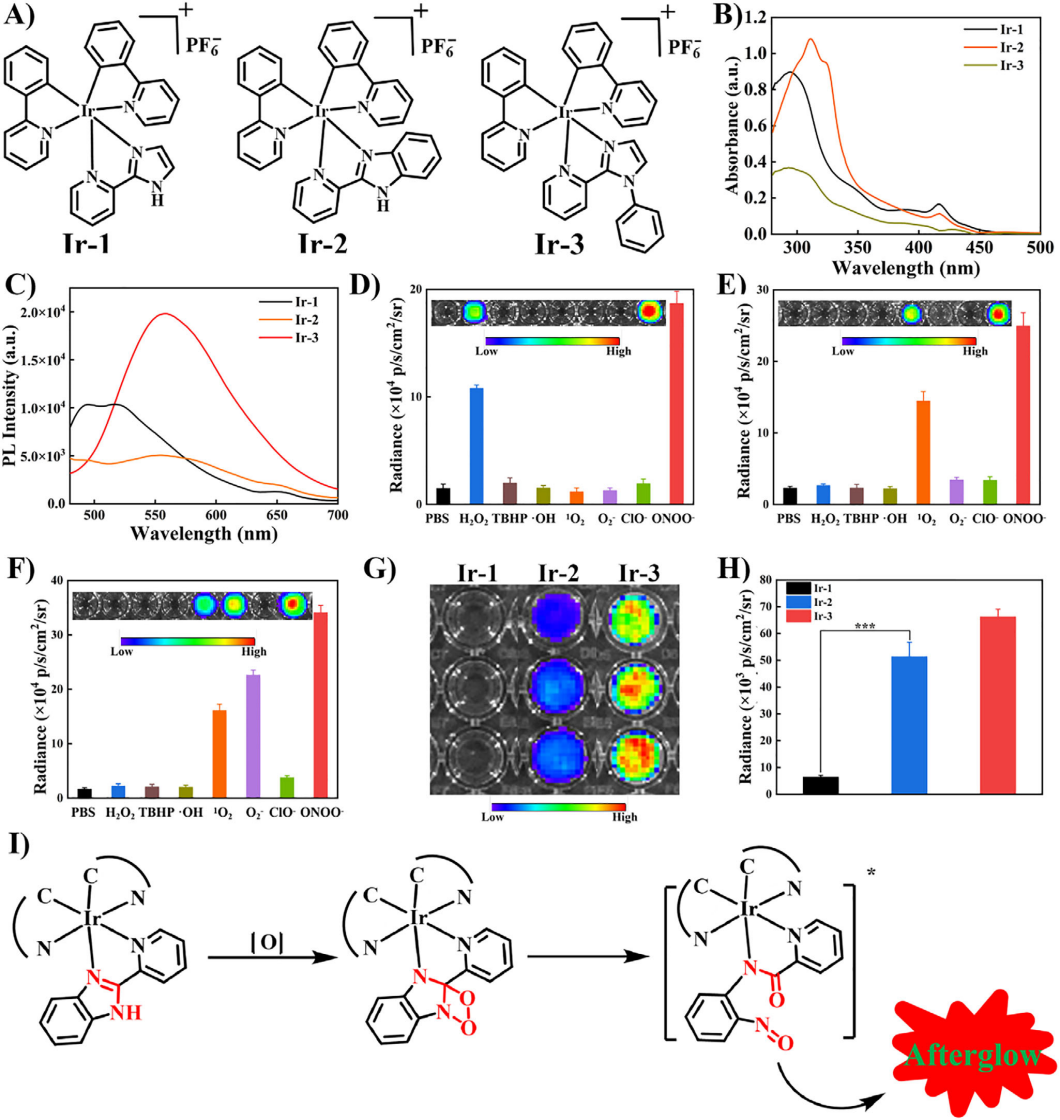
(A) The structural formulas of Ir‐1, Ir‐2, and Ir‐3. (B) UV–vis absorption spectra of Ir‐1, Ir‐2, and Ir‐3 in tetrahydrofuran (THF) (complex concentration = 1.0 ×10^−5^ M). (C) Fluorescence spectra of Ir‐1, Ir‐2, and Ir‐3 in THF (complex concentration = 1.0 ×10^−5^ M) under 365 nm UV irradiation. Afterglow luminescence images of (D) Ir‐1, (E) Ir‐2, and (F) Ir‐3 after addition of PBS and different RONS (100 µM for each). Inset: the afterglow luminescence images acquired by an IVIS imaging system under bioluminescence mode with an open filter. (G) Afterglow luminescence images of Ir‐1, Ir‐2, and Ir‐3 (100 µM) in THF after cessation of US pre‐irradiation (1.0 W cm^−2^, 1 MHz, 50% duty cycle). (H) The quantification of corresponding afterglow luminescence intensities in (G). (I) Proposed mechanism for the generation of afterglow luminescence. Data are represented as mean ± SD (*n* = 3). The data were analyzed by unpaired 2‐tailed Student's *t*‐test using the GraphPad Prism 7. *p < 0.05, **p < 0.01, ***p < 0.001.

Turning to potential biomedical applications of the Ir complexes, we note that critical vascular diseases constitute an important cause of global morbidity and mortality for humanity [50, 51]. Timely and accurate detection of these injuries enables prompt therapeutic intervention which is of vital importance for preventing irreversible damage and improving the prognosis for patients. Peroxynitrite (ONOO^−^) is a RONS and the overexpression of ONOO^−^ is known to be involved in the occurrence and development of various diseases, including inflammation, ischemia‐reperfusion injury, and cardiovascular diseases [52, 53]. Therefore, by using imidazole derivatives as the framework for the afterglow luminescence, we aimed to develop highly efficient ONOO^−^‐activated near‐infrared afterglow luminescence probes. Through afterglow luminescence imaging, conducting the in situ visual detection of ONOO^−^ levels in thrombotic diseases will greatly facilitate the accurate and early diagnosis of vascular injuries.

In this work, we have now designed and synthesized a series of Ir(III) complexes with common, cheap, and commercially available imidazole derivative N^N ligands, named Ir‐X (X = 1–4; structures are shown in Figures 1, 2, 3, 4). The complexes can respond to US irradiation or ONOO^−^ to selectively activate near‐infrared afterglow luminescence, thereby enabling excellent signal‐to‐background ratio imaging and detection in vitro. Among them, Ir‐4 has coordinated quinolyl‐triphenylamine C^N ligands which not only endows the derived nanoparticles Ir‐4@R NPs with AIE properties but also enables the construction of a near‐infrared afterglow luminescent molecule. In vitro experiments, establish that Ir‐4 has excellent sonodynamic capabilities and emits bright near‐infrared afterglow luminescence under the stimulation of ONOO^−^ or ultrasound excitation. Moreover, in vivo experiments achieved precise near‐infrared afterglow luminescence targeted imaging by endogenous RONS and restored blood flow in a mouse carotid artery. Our research not only reveals a novel mechanism for afterglow luminescence in imidazole derivatives but also offers a new approach for the design of metal complexes that show afterglow luminescence.

**FIGURE 4:**
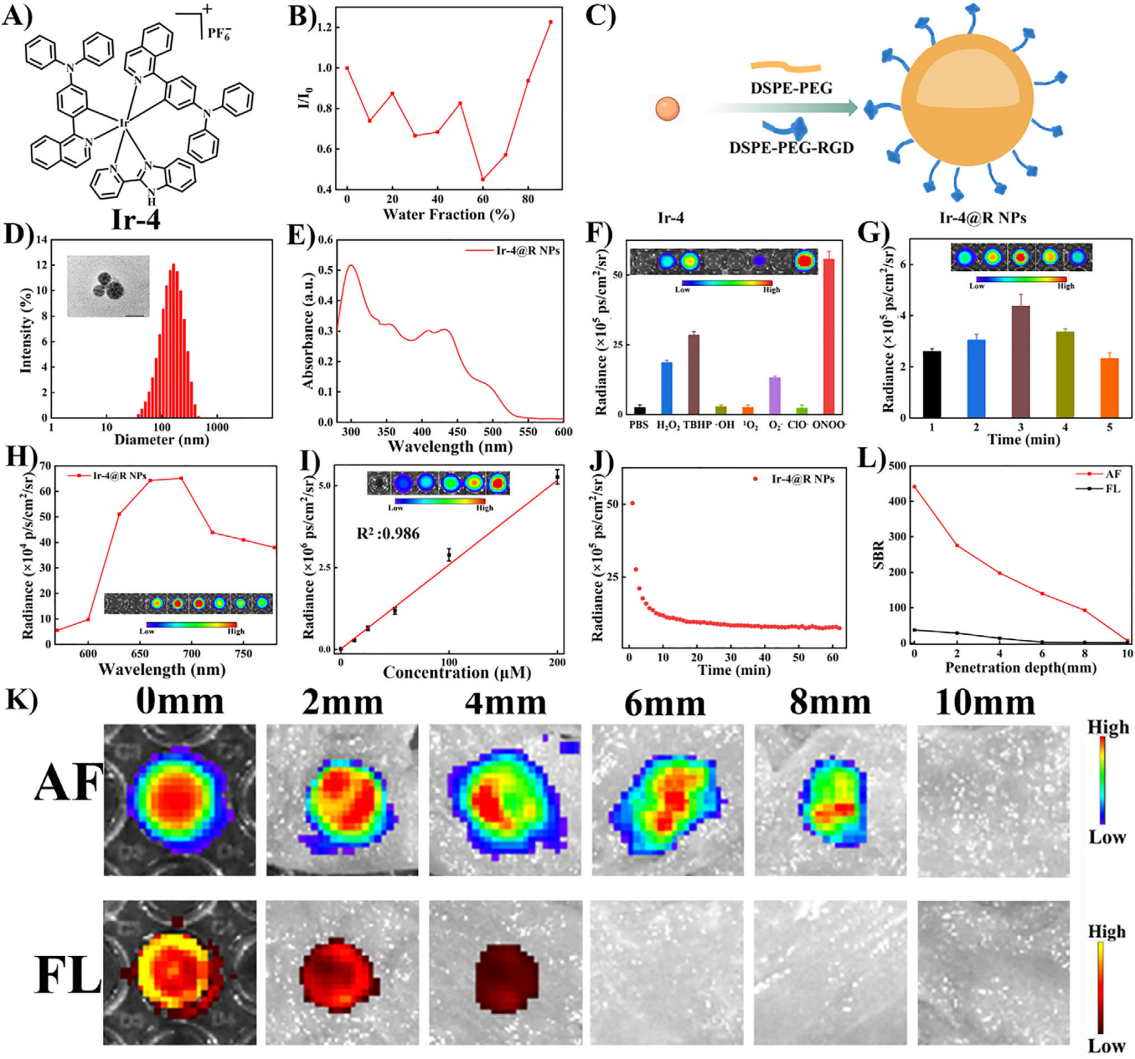
(A) The structural formulas of Ir‐4. (B) Fluorescence of Ir‐4 in methanol and different methanol:water fractions (complex concentration = 1.0 ×10^−5^ M). (C) The preparation process of Ir‐4@R NPs. (D) DLS diagram of Ir‐4@R NPs, inset: the TEM image of Ir‐4@R NPs. Scale bar: 200 µm. (E) UV–vis absorption spectra of Ir‐4@R NPs in water. (F) Afterglow luminescence images of Ir‐4@R NPs after addition of PBS and different RONS (100 µM for each). Inset: the afterglow luminescence images acquired by an IVIS imaging system under bioluminescence mode with an open filter. (G) Afterglow luminescence images of Ir‐4@R NPs (100 µg/mL) after cessation of US pre‐irradiation (1.0 W cm^−2^, 1 MHz, 50% duty cycle). Insets: the corresponding afterglow luminescence acquired on an IVIS imaging system. (H) Afterglow luminescence spectra of Ir‐4@R NPs in the absence or presence of ONOO^−^ (100 µM). Insets: the corresponding afterglow luminescence acquired on an IVIS imaging system. (I) Afterglow luminescence intensities of Ir‐4@R NPs after addition of ONOO^−^. Insets: the corresponding afterglow luminescence acquired on an IVIS imaging system. (J) Decay of afterglow luminescence signal of Ir‐4@R NPs over time at room temperature after addition of ONOO^−^. (K) Tissue penetration depths after the activated afterglow luminescence of Ir‐4@R NPs + ONOO^−^ and the NIR fluorescence from Ir‐4@R NPs with a coverage of chicken breast tissues with different thicknesses. (L) Signal‐to‐background ratios (SBRs) for afterglow luminescence or FL images as a function of chicken tissue depth. Data are represented as mean ± SD (*n* = 3).

## 2 Results and Discussions

We initially observed that a mixture of L2 and ONOO^−^ (pH = 7.4) gave no afterglow luminescence. However, when the derived Ir(III) complex Ir‐2 was mixed with ONOO^−^, bright afterglow luminescence was observed (Figure 2A). This result clearly indicates that the construction of Ir(III) complex activates the ─N═C─N─H site of the imidazole, thereby enabling them to react with RONS to form high‐energy intermediates and turn‐on the afterglow luminescence. This experimental observation was verified through density functional theory (DFT) calculations shown in Figure 2B. The calculated energy gap Δ*E*_gap_ (*E*_LUMO_−*E*_HOMO_) for L2 and Ir‐2 was 4.85 and 3.47 eV, respectively, demonstrating that the iridium complexation effectively reduces the HOMO–LUMO excitation energy, which endows the compound with high reactivity. Figure 2C shows that the energy barrier of Ir‐2 is 15.93 kcal mol^−1^, which is significantly lower than that of L2 (24.85 kcal mol^−1^). Hence Ir‐2 is more likely to react with ONOO^−^ to form a high‐energy intermediate for the afterglow luminescence.

Based on the aforementioned encouraging experimental results, and taking advantage of the adjustable structure of Ir(III) complexes, phenylpyridine was selected as the C^N ligands, and two imidazolylpyridine derivatives were introduced as the N^N ligands to obtain the analogous complexes Ir‐1 and Ir‐3. The synthetic routes (Schemes S1–S3) and corresponding characterization data of ligand L3, complexes Ir‐1–Ir‐3 are presented along with ^1^H NMR and mass spectrometry data (Figures S1–S6). The structural formulas of the three Ir(III) complexes with afterglow luminescence are shown in Figure 3A. The ultraviolet–visible (UV–vis) absorption and fluorescence spectra of the complexes were studied in tetrahydrofuran (THF) solution. They all have strong absorption peaks in the ultraviolet region, and the bands in the range of 280–350 nm are attributed to the π→π* transition centered on the ligands. The relatively weak absorption bands in the visible region can be attributed to combined metal‐to‐ligand charge transfer transitions, spin‐permitted ligand‐to‐ligand charge transfer transitions, spin‐barred metal‐to‐ligand charge transfer transitions, and spin‐barred ligand‐to‐ligand charge transfer transitions [54, 55]. As shown in Figure 3C, Ir‐1, Ir‐2, and Ir‐3 have an emission peak at about 522, 554, and 558 nm, respectively.

The selective afterglow luminescence of Ir‐1–Ir‐3 was investigated using phosphate‐buffered saline (PBS), reactive oxygen and nitrogen species (RONS) including *t*‐Bu‐hydroperoxide (TBHP), •OH, O_2_^−^, ^1^O_2_, H_2_O_2_, ClO^−^, and ONOO^−^. Ir‐1, Ir‐2, and Ir‐3 showed a degree of response to some of these species. Interestingly, ONOO^−^ was the most efficient afterglow luminescence initiator for all the three complexes Ir‐1, Ir‐2 and Ir‐3, showing activated afterglow luminescence intensity much higher than PBS or the other RONS (Figure 3D–F).

Subsequently, the sonoafterglow properties of Ir‐1, Ir‐2, and Ir‐3 were studied using an in vivo imaging system (IVIS). After 3‐min stimulation with ultrasound (1.0 W cm^−2^, 1 MHz, 50% duty cycle), Ir‐1 did not exhibit afterglow luminescence, whereas Ir‐2 and Ir‐3 possessed clear sonoafterglow luminescence, which was brightest for Ir‐3 (Figure 3G,H). The reaction sites of most of the reported afterglow luminescent frameworks are C═C double bond structures [22, 28]. We believe that the reaction site of the imidazole derivative ligand in Ir‐2 is distinctive from the reaction at the ─C═C─ unit of the chemiluminescent diphenylimidazole derivatives proposed by Yang et al. [39]. The suggested oxidative reaction mechanism for Ir‐2 is shown in Figure 3I where the ─N═C─N─H unit is the reactive site for ONOO^−^. The derived high‐energy dioxetane intermediate generates triplet excited states of the complex, and their subsequent relaxation to ground states is accompanied by the observed emission. These results were further supported by the mass spectrometric detection of the reaction product (Figure S9). However, there is scope for further studies on the afterglow luminescence mechanism of this reaction reported here for the first time.

Based on the aforementioned successful experiments, we introduced the bulky quinolyl‐triphenylamine C^N ligand and constructed the Ir(III) complex probe with AIE properties based on our previous work (Figure 4A) [56, 57]. The synthetic route (Scheme S4) and corresponding ^1^H NMR and mass spectrometry characterization data of complex Ir‐4 are presented in Figures S7 and S8. As shown in Figure S10A, Ir‐4 has a strong emission peak in methanol solution at about 626 nm in the NIR region. Figures 4B and S10B show that Ir‐4 exhibits weak emission in methanol, but in a solution with water content of 90%, its fluorescence intensity significantly increased, which is typical AIE behavior. This result indicates that the ROS production capacity of Ir‐4 will not be weakened when it is Ir‐4@R NPs. Subsequently, Ir‐4 was assembled into water‐soluble nanoparticles through the classical nanoprecipitation method with DSPE‐PEG and DSPE‐PEG‐RGD as the surfactant (Figure 4C). To endow the nanoparticles with thrombus‐targeting ability, the peptide c(RGDfC) was attached to the NPs, thereby forming Ir‐4@R NPs. Dynamic light scattering (DLS) established that the hydrodynamic diameter of the Ir‐4@R NPs was 164 nm (Figure 4D). Transmission electron microscopy (TEM) revealed that Ir‐4@R NPs have a uniform spherical shape and an average size of 143 nm (Figure 4D inset). The zeta potential at the site of the nanoparticles after modification with the targeting peptide changed from 4.1 mV to −11.5 mV (Figure S11). As depicted in Figure S12, Ir‐4@R NPs showed good colloidal stability after 7 days of storage in aqueous solution. The UV–vis‐NIR absorption spectrum of Ir‐4@R NPs shows a longest absorption wavelength at approximately 550 nm (Figure 4E).

PBS, *t*‐Bu‐hydroperoxide (thbhp), •OH, O_2_^−^, ^1^O_2_, H_2_O_2_, ClO^−^, and ONOO^−^ were investigated as RONS to assess the selectivity of the afterglow luminescence of Ir‐4@R NPs. Similarly to Ir‐1, Ir‐2, and Ir‐3 discussed above, ONOO^−^ was the most efficient initiator for Ir‐4@R NPs afterglow luminescence (Figure 4F). Furthermore, it is remarkable that the afterglow luminescence intensity of Ir‐4 NPs was enhanced by more than 20‐fold compared to Ir‐2. Based on this, we compared the afterglow luminescence of Ir‐2 and Ir‐4 and found that the afterglow luminescence of Ir‐4 was four times greater than that of Ir‐2 (Figure S13). This is attributed to the introduction of the quinoline triphenylamine ligand in Ir‐4. The reason why the afterglow luminescence is further enhanced after Ir‐4 was prepared into nanoparticles is that when Ir‐4 is in the AIE state, it has higher fluorescence quantum yield which will further increase the intensity of its chemiluminescence/afterglow luminescence [35]. Subsequently, we studied the sonoafterglow properties of Ir‐4@R NPs. The results showed that Ir‐4@R NPs exhibit bright afterglow luminescence under ultrasound (1.0 W cm^−2^, 1 MHz, 50% duty cycle), and afterglow luminescence intensity was the strongest after continuous ultrasound excitation for 3 min (Figure 4G). We investigated the reproducibility of the afterglow luminescence of Ir‐4@R NPs. Three groups of Ir‐4@R NPs were separately prepared for US response and ONOO^−^ response (Figure S14). The afterglow luminescence intensities of the different repeat groups under US or ONOO^−^ conditions were basically the same. These results demonstrate that when the single‐molecule afterglow luminescence probe Ir‐4 is prepared as nanoparticles, it exhibits excellent reproducibility. The afterglow luminescence peak of Ir‐4@R NPs was at approximately 680 nm (Figure 4H). This proves the successful construction of an AIE Ir(III) complex with bright near‐infrared afterglow luminescence. The afterglow luminescence signal of Ir‐4@R NPs has a very good linear correlation with ONOO^−^ concentration, including a limit of detection (LOD) of 45.3 nM, calculated from 3*σ*/*k* (*σ* is the standard deviation of blank tests; *k* is the slope of the working curve) (Figure 4I). The excellent detection sensitivity and selectivity enable the probe to achieve precise in vivo imaging. In addition, it can be observed that Ir‐4@R NPs retain good residual afterglow luminescence for up to 5 min, which is suitable for in vivo imaging (Figure 4J).

We investigated the penetration depth of the afterglow luminescence imaging of Ir‐4@R NPs. Chicken breast tissue slices of different thickness were placed on the sample and ONOO^−^ was added to induce the afterglow luminescence of the Ir‐4@R NPs. Without the presence of chicken tissue, the imaging showed extremely low background noise with an SBR of 441.52, which was approximately 12‐fold higher than that of fluorescence (FL) (36.1) (Figure 4K,L). Both the afterglow luminescence and FL signal of Ir‐4@R NPs decreased with increasing thickness of the chicken tissue (Figure 4K,L). Interestingly, due to the poor penetration ability of the excitation light and the significant background interference, when the chicken tissue reached a thickness of 6 mm, the FL signal of the Ir‐4@R NPs almost disappeared. However, the afterglow luminescence signals can still be clearly detected. As afterglow luminescence possesses a very low background noise, its SBR is as high as 92.49 at 8 mm thickness, whereas the FL of Ir‐4@R NPs is close to the tissue autofluorescence background (Figure 4K,L). These results demonstrate that the excellent near‐infrared afterglow luminescence exhibited by Ir‐4@R NPs can eliminate the noise of tissue spontaneous fluorescence, which is beneficial for improving the imaging depth and SBR of in vivo imaging.

We next investigated the ROS generating ability of Ir‐4@R NPs which is of great significance for sonoafterglow luminescence and thrombosis treatment (Figure 5A). Using 1,3‐diphenylisobenzofuran (DPBF) as a general ROS scavenger [58], the absorption spectrum of DPBF continuously decreased under ultrasonic irradiation (1.0 W cm^−2^, 1 MHz, 50% duty cycle). As shown in Figure 5B–D, after 15 min of ultrasound irradiation, the absorption at 420 nm in the Ir‐4@R NPs decreased by 60%. However, in the control experiment without Ir‐4@R NPs, the absorption band of DPBF decreased by only about 5%. These results proved that Ir‐4@R NPs are an excellent sonodynamic nanoplatform. We then explored the in vitro tissue penetration ability of ultrasound. Subsequently, by investigating the absorption signals at 420 nm of DPBF covered with 2, 4, and 6 mm of chicken breast tissue, the extent of tissue penetration under ultrasonic irradiation (1.0 W cm^−2^, 1 MHz, 50% duty cycle) and white light irradiation (20 mW cm^−2^) were compared. Figure 5E,H showed that when covered with 2 mm of chicken breast meat, the DPBF absorption signal decreased by approximately 20% in the light irradiation group. However, when covered with 4 or 6 mm thick chicken breasts, the DPBF absorption signal of the light irradiation group decreased extremely slowly because light cannot effectively penetrate the chicken breast tissue to stimulate Ir‐4@R NPs to generate singlet oxygen (Figure 5F–H). In contrast, the DPBF absorption signal of the ultrasound group significantly decreased with time with 2, 4, or 6 mm thick chicken breasts (Figure 5I–L). Ultrasound retained nearly 70% of its effectiveness after penetrating 2 mm thick chicken breast. Even when covered with 6 mm thickness of chicken breast, ultrasound is still able to activate Ir‐4@R NPs to maintain sonodynamic efficiency of around 40%. There results showed that ultrasonic energy can be effectively delivered into deep tissue and replace light as an excellent irradiation source.

**FIGURE 5:**
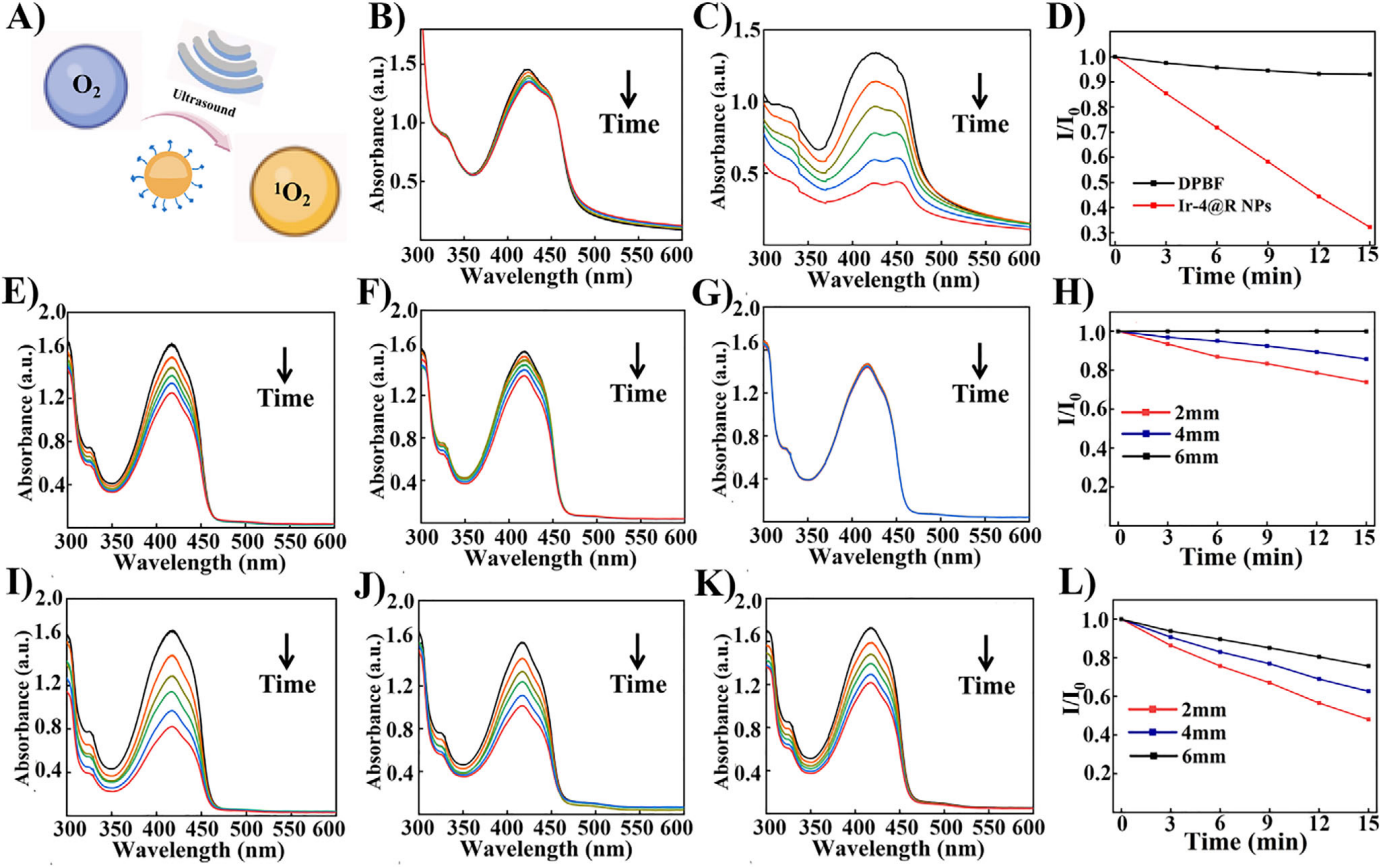
(A) Schematic diagram of ultrasound‐induced production of ROS by Ir‐4@R NPs. (B) UV–vis absorption spectra of DPBF under ultrasound irradiation (1.0 W cm^−2^, 1 MHz, 50% duty cycle). (C) UV–vis absorption spectra of DPBF in the presence of Ir‐4@R NPs under ultrasound irradiation (1.0 W cm^−2^, 1 MHz, 50% duty cycle). (D) Comparison of the decay rates at different conditions under ultrasound irradiation (1.0 W cm^−2^, 1 MHz, 50% duty cycle), *I*_0_ = absorption of DPBF without irradiation. *I* = real‐time absorption of DPBF with different irradiation time; UV–vis absorption spectra changes of the reaction mixture under white light irradiation (20 mW cm^−2^) at different time intervals covered with (E) 2 mm chicken breast tissues, (F) 4 mm chicken breast tissues, (G) 6 mm chicken breast tissues, (H) comparison of the decay rates at different chicken breast tissues under light irradiation (20 mW cm^−2^), *I*_0_ = absorption of DPBF without irradiation. *I* = real‐time absorption of DPBF with different irradiation time; UV–vis absorption spectra of the reaction mixture under white light irradiation (20 mW cm^−2^) at different time intervals covered with (I) 2 mm chicken breast tissues, (J) 4 mm chicken breast tissues, (K) 6 mm chicken breast tissues; (L) comparison of the decay rates at different chicken breast tissues under ultrasound irradiation (1.0 W cm^−2^, 1 MHz, 50% duty cycle), *I*_0_ = absorption of DPBF without irradiation. *I* = real‐time absorption of DPBF with different irradiation time.

Excellent ROS generation ability is a key requirement for extracorporeal thrombolysis. First, the biocompatibility of Ir‐4@R NPs was evaluated through hemolysis experiments (Figure 6A). With the concentrations of Ir‐4@R NPs ranging from 0 µg mL^−1^ to 200 µg mL^−1^, all the blood samples showed relatively low hemolysis ratios of <5%, indicating the good biocompatibility of the Ir‐4@R NPs. The targeting specificity of Ir‐4@R NPs for thrombi was investigated through in vitro targeted blood clot experiments. Fresh mouse blood was mixed with the coagulant to prepare artificial blood clots. Then Ir‐4 NPs (without c(RGDfC) targeting peptide) and Ir‐4@R NPs were incubated for 1, 3, or 5 h, respectively. After this time, the clots were visualized with an in vivo imaging system (IVIS). As expected, the blood clots incubated with Ir‐4@R NPs exhibited a remarkably stronger fluorescent signal than Ir‐4 NPs and PBS groups at each time point (Figure 6B,C) due the improved targeting mediated by the c(RGDfC) peptide component in the Ir‐4@R NPs. Then, different treatments were applied to the blood clots, namely PBS, ultrasound (US), urokinase (UK), and Ir‐4@R NPs + ultrasound to evaluate their thrombolytic effects. The blood clots in all groups were treated for 90 min. The blood clots in the Ir‐4@R NPs + ultrasound group shrank significantly, and the supernatant was blood red (Figure 6D,E). In contrast, the blood clots in the other groups showed only slight or moderate dissolution.

**FIGURE 6:**
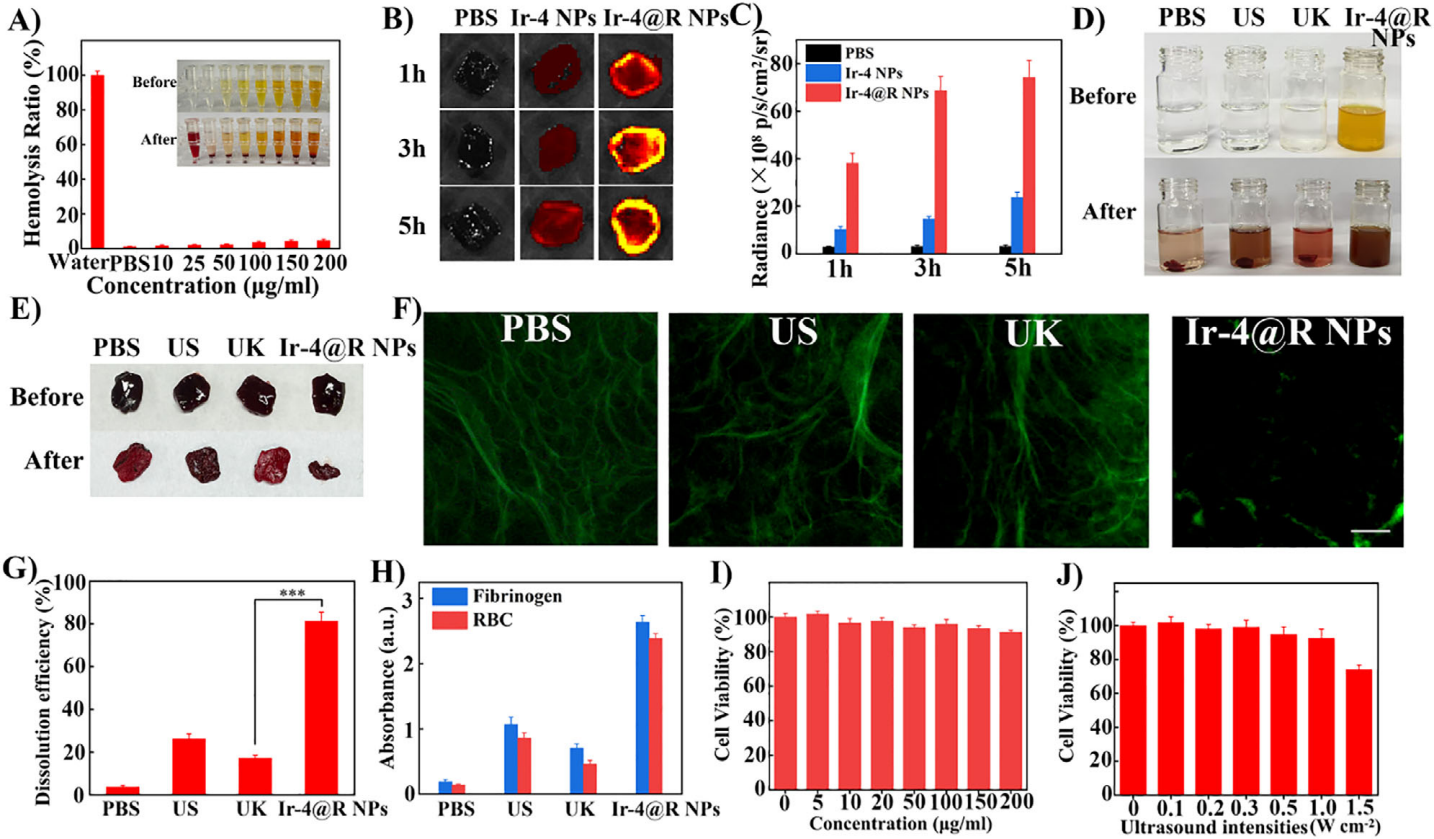
(A) The hemolysis ratio of red blood cells treated with water, PBS, and different concentrations of Ir‐4@R NPs in water. Inset shows the red blood cells solution upon different treatments (from left to right: water, PBS, 10, 25, 50, 100, 150, and 200 µg mL^‒1^ of Ir‐4@R NPs). (B) The fluorescent signal of artificial blood clots after incubation with PBS, Ir‐4 NPs, or Ir‐4@R NPs. (C) Quantitative analysis of the relative persistent intensity of the artificial blood clots in (B). (D and E) Photographs of the blood clot solution after different treatments (from left to right: PBS, ultrasound, free UK, Ir‐4@R NPs + ultrasound). The residual blood clots were taken out and shown. (F) Fluorescence images of fibrin clots with different treatments (from left to right: PBS, US, UK, and 200 µg mL^‒1^ of Ir‐4@R NPs + ultrasound). Scale bar: 100 µm. (G) thrombolytic rates were quantitatively analyzed by measuring the percentage of thrombus mass loss after different treatments in (E). (H) The absorbance at 450 nm (blue) and 540 nm (red) of the supernatant in different groups (PBS, US, UK, Ir‐4@R + US) after 60 min of treatment. (I) The cytotoxicity of different concentrations of Ir‐4@R NPs in HUVECs. (J) The cytotoxicity of 10 min of ultrasound at different intensities in HUVECs. Data are represented as mean ± SD (*n* = 3). The data were analyzed by unpaired 2‐tailed Student's *t*‐test using the GraphPad Prism 7. *p < 0.05, **p < 0.01, ***p < 0.001.

To further investigate the destructive effect of ROS on the fibronectin scaffold in thrombi, we labeled fibrin with fluorescein isothiocyanate (FITC) and then observed the fluorescence intensity and extent of collapse of the fibrin scaffold after treatment in different groups. The fibrin framework in the Ir‐4@R NPs + ultrasound group was significantly disrupted. In contrast, in the ultrasound only or free UK groups, obvious green fluorescence and an intact framework structure could still be observed. This indicates that the excellent ROS generation of Ir‐4@R NPs, to disrupt the fibrin clot, originated from the AIE performance of the complex (Figure 6F). The thrombolytic efficiency was quantitatively analyzed by measuring the percentage of thrombus mass loss after different treatments. The thrombolysis rates of the PBS, US, UK, and Ir‐4@R NPs + ultrasound groups were 4.7%, 28.3%, 17.3%, and 81.4%, respectively (Figure 6G). Compared with the UK group, the absorbance of the supernatant in the Ir‐4@R NPs + ultrasound group significantly increased at 450 and 540 nm, indicating that a large amount of fibrin and hemoglobin had dissociated after thrombolysis (Figure 6H). Clearly, the Ir‐4@R NPs + ultrasound group had the strongest in vitro thrombolysis ability. These results indicate that the effect of Ir‐4@R NPs + ultrasound was much better than that of the drug UK.

The cytotoxicity of Ir‐4@R NPs on human umbilical vein endothelial cells (HUVECs) was evaluated. Ir‐4@R NPs were co‐cultured with HUVECs for 24 h. Then the cells’ viability was analyzed using 3‐(4,5‐dimethylthiazol‐2‐yl)‐2,5‐diphenyltetrazolium bromide (MTT) assay, which showed that >90% of the cells remained alive after treating with 0–200 µg mL^‒1^ of Ir‐4@R NPs demonstrating their negligible cytotoxicity (Figure 6I). Then, the effects of different ultrasonic intensities on the viability of HUVECs were investigated. When HUVECs were treated with ultrasonic waves at an intensity of 0.1–1.0 W cm^−2^ for 10 min, the cells’ viability was almost unaffected. However, the cells showed significant death at an intensity of 1.5 W cm^−2^ (Figure 6J). Therefore, subsequent experiments used ultrasonic intensity of 1.0 W cm^−2^.

Afterglow luminescence effectively eliminates the fluorescence noise generated by the tissue itself which is beneficial for deep tissue imaging and signal‐to‐background ratio (SBR) imaging in vivo. Accurate thrombus‐targeted imaging is the key to the success of thrombolysis using sonodynamic therapy. After intravenous (tail vein) injection of Ir‐4@R NPs (5 mg/kg) into mice, when Ir‐4@R NPs target and accumulate at the thrombus site, they will react with the overexpressed H_2_O_2_ and ONOO^−^ at the thrombus site to release afterglow luminescence; real‐time afterglow luminescence images were captured by an IVIS system. Compared with the normal group, a significant afterglow luminescence signal was observed at the thrombus site in the Ir‐4@R NPs group (Figure 7A,B). The signal reached its peak at 60 min, demonstrating that Ir‐4@R NPs remained in the blood vessels for a relatively long time. Subsequently, the sonodynamic thrombolytic therapy of Ir‐4@R NPs was investigated. The therapeutic effects of different treatment protocols on a mouse carotid artery thrombosis model were evaluated. A Laser Speckle Blood Flow Monitoring System (LSBFMS) captured the hemodynamic changes in the blood vessels in real time for imaging and quantification. First, the changes in blood after the induction of a carotid artery thrombus model were analyzed. After exposure of the carotid artery to 10% aqueous FeCl_3_ solution for 5 min, the blood flow in the model vessel was dramatically retarded, and a complete and stable vascular embolism was formed after 10 min. Then different formulations, including PBS, free UK and Ir‐4@R NPs (5 mg/kg), were separately administrated into the mouse via the tail vein. In the UK treatment group, free UK was injected into the mice via the tail vein at a dose of 0.04 mg/kg. The free UK travelled with the blood flow to the thrombus site and dissolved the blood clots, restoring the blood flow to a certain extent. However, due to the short lifespan of large‐molecule protein drugs in the blood, UK was unable to completely re‐open the blocked blood vessels, resulting in another blockage 30 min later (Figure 7C,D). Notably, the Ir‐4@R NPs group possesses both thrombus‐targeting properties and the therapeutic effect of sonodynamic therapy under ultrasound irradiation, which showed significant effective thrombus dissolution and the blood flow recovered to approximately 71% (Figure 7C,D). These results indicate that the SDT significantly promotes thrombus therapy. The mouse carotid arteries were collected for hematoxylin‐eosin (H&E) staining to assess the therapeutic efficacy by measuring the thrombus area (Figure 7E). The Ir‐4@R NPs group successfully dissolved most of the blood clots in the blood vessels. To assess whether the Ir‐4@R NPs thrombolysis treatment had a risk of hemorrhagic side effects the mice were first intravenously injected with PBS, free UK, and Ir‐4@R NPs, and then their tails were cut with a scalpel and the bleeding time was monitored. For the free UK group, the bleeding time significantly increased (about 20.3 min), which was four‐fold longer than that of the PBS‐injected group (about 5.1 min). The Ir‐4@R NPs group displayed a similar bleeding time to the PBS group (around 5.9 min) and similar bleeding volume (Figure S15). These data prove that Ir‐4@R NPs exhibited a good biosafety profile without the high bleeding risk of UK.

**FIGURE 7:**
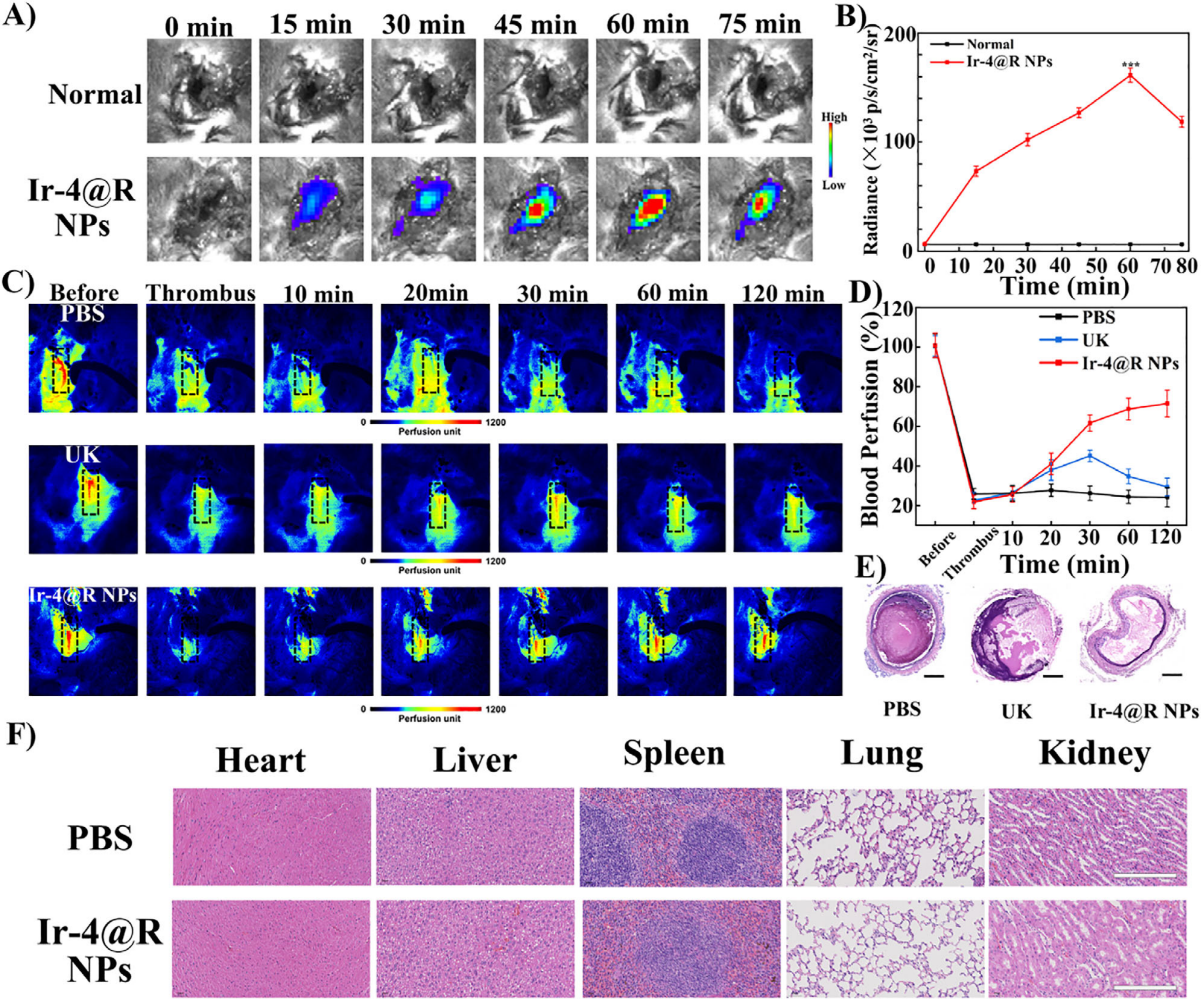
(A) IVIS images of carotid artery site in thrombus mouse and normal mouse post‐injection of Ir‐4@R NPs (5 mg/kg). (B) In vivo persistent intensity curves of carotid artery site in thrombus mouse and normal mouse post‐injection of Ir‐4@R NPs (5 mg/kg). (C) Representative LSBFMS analysis of the mouse carotid artery after FeCl_3_ induction and different therapeutic treatments with PBS, free UK, and Ir‐4@R NPs + ultrasound; the dashed black boxes indicate the ROI corresponding to the data presented in Figure 7D). (D) The corresponding relative blood perfusion of the mouse carotid artery after FeCl_3_ induction and different therapeutic treatments with PBS, free UK, and Ir‐4@R NPs + ultrasound. (E) Hematoxylin–eosin staining evaluated the thrombus resolution in the carotid artery following various treatments. Scale bar: 200 µm. (F) H&E staining of various organs from mice after different treatments. Representative H&E‐stained images from heart, liver, spleen, lung, and kidney slices. Scale bars = 200 µm. Data are represented as mean ± SD (*n* = 3). The data were analyzed by unpaired 2‐tailed Student's *t*‐test using the GraphPad Prism 7 (B). *p < 0.05, **p < 0.01, ***p < 0.001.

Finally, blood tests showed that the hematological indexes of the mice treated with Ir‐4@R NPs were within the normal range (Figure S16). Moreover, multiple serum biochemical markers for liver and renal damage, including blood urea nitrogen (BUN), creatinine (CREA), alanine aminotransferase (ALT), and aspartate aminotransferase (AST) were measured for analyzing the liver and kidney functions, as shown in Figure S17. The histological images of all the organs showed no detectable pathological changes or inflammatory reactions after treatment with Ir‐4@R NPs (Figure 7F). These hepatorenal indicators showed no significant differences between PBS and Ir‐4@R NPs‐treated mice. Meanwhile, the biocompatibility of Ir‐4@R NPs was evaluated. By day 7, Ir‐4@R NPs were mainly metabolized by the liver and kidneys. In the organ fluorescence imaging on day 7, almost no fluorescence signals were detected which proved that the Ir‐4@R NPs had been almost completely metabolized. (Figure S18). All the experiments fully demonstrate that Ir‐4@R NPs have good biosafety in vivo, which is an important prerequisite for their future clinical applications.

## 3 Conclusion

In summary, we report for the first time a series of novel iridium(III) complexes that exhibit unexpected stimulus‐responsive near‐infrared afterglow luminescence through coordination with imidazole derivatives. Further research has proposed a plausible mechanism for the afterglow luminescence, which involves oxidation at ─N═C─N─H sites in the imidazole unit, as evidenced by mass spectrometry and theoretical calculations of the reaction products of the afterglow luminescence. Subsequently, we prepared Ir‐4 with AIE properties by modifying the structure of the C^N ligands. After encapsulating Ir‐4 into nanoparticles, the afterglow luminescence intensity was enhanced by more than 20‐fold compared to Ir‐2. Under neutral conditions, Ir‐4 can respond to various reactive oxygen and reactive nitrogen species and exhibit a strong afterglow luminescence reaction. Finally, Ir‐4@R NPs demonstrated excellent afterglow luminescence imaging and sonodynamic therapy effects in a mouse carotid artery thrombus inflammation model, highlighting the excellent biocompatibility and feasibility of Ir‐4@R NPs as an afterglow luminescence biological probe. This study showcases the application of extremely common and inexpensive imidazole derivatives as afterglow luminescent materials and also expands the library of molecular frameworks for in vivo imaging based on afterglow luminescence.

## Author Contributions

**Zihan Wu**: writing – original draft, writing – review and editing, investigation, conceptualization, methodology, validation, data curation. **Liping Zhang**: validation, writing – review and editing. **Ziwei Wang**: validation. **Yachao Wang**: validation. **Gelin Xu**: validation. **Feng Chi**: validation. **Martin R. Bryce**: writing – review and editing, data curation, methodology, funding acquisition. **Dongxia Zhu**: data curation, methodology, funding acquisition. **Lijie Ren**: supervision, funding acquisition. **Ben Zhong Tang**: validation.

## Conflicts of Interest

The authors declare no conflicts of interest.

## Supporting information




**Supporting File**: anie74505‐sup‐0001‐SuppMat.pdf

## Data Availability

The data that support the findings of this study are available in the Supporting Information file.
