# Supplementary material for: Created Ex Nihilo: A Near‐Infrared Afterglow Luminescent Framework of Imidazole Derivatives for Bioimaging
Source: Angew Chem Int Ed Engl. 2026 Sep 2;65(43):e9929915. doi: 10.1002/anie.9929915 (PMC13676407; doi:10.1002/anie.9929915)
Supplement: Supplementary file 1 [file ANIE-65-e9929915-s001.pdf]

Supporting Information

General Materials and Methods.

All the chemicals were obtained from Sigma-Aldrich unless otherwise specified. The solvents for chemical reactions were distilled before use. Materials for organic synthesis were purchased from Energy Chemical Company. 1,2-Distearoyl-sn-glycero-3-phosphoethanolamine-polyethylene glycol-RGD(ARG-GLY-ASP) was purchased from Laysan Bio, Inc. (Arab, AL). c(RGDfC) was supplied by Hangzhou Special Peptide Biotechnology Co. 1,3-Diphenylisobenzofuran (DPBF) was purchased from Energy Chemical Company. Urokinase (UK, 1.2 × 105 IU/mg) was obtained from Aladdin Biochemical Technology Co, Ltd. (Shanghai, China). 3-(4,5-dimethyl-2-thiazolyl)-2,5-diphenyl-2*H*-tetrazolium bromide (MTT) was purchased from Shanghai Beyotime Biotechnology Co. Ltd. Enzyme-linked immunosorbent assay (ELISA) kit was provided by Biolegend.

Reactions were monitored with analytical thin-layer chromatography (TLC) on silica. ^{1}H NMR spectra were recorded at 25 °C on a Varian 500 MHz spectrometer. Mass spectra were recorded on a Bruker autoFlex III instrument. UV-vis absorption spectra were recorded on a Shimadzu UV-3100 spectrophotometer. The photoluminescence spectra were recorded on an Edinburgh FLS920 spectrofluorimeter under air at room temperature. Transmission electron microscopy (TEM) images were taken by a TECNAI F20 microscope. Diameter and diameter distribution of the nanoparticles were determined by a Malvern Zetasizer Nano instrument for dynamic light scattering (DLS). Confocal laser scanning microscopy (CLSM) images were taken using a LSM 800 Zeiss, Germany. Chemiluminescence and fluorescence data were acquired by an in vivo imaging system (Xenogen IVIS Lumina II system).

Quantum chemical calculations.

All quantum chemical calculations were performed using the ORCA program package.[1] Geometry optimizations and vibrational frequency analyses were carried out at the PBE0-D3(BJ)/def2-SVP level of theory,[2-4] and the absence of imaginary frequencies was confirmed to ensure that the optimized structures correspond to true minima on the potential energy surface. Molecular orbitals were visualized using the VESTA program.[5] Transition states were located at the same level of theory, and each was verified by the presence of one and only one imaginary frequency, corresponding to a first-order saddle point on the potential energy surface. To obtain more accurate electronic energies, single-point energy calculations were performed at the PWPB95-D4/def2-QZVPP level.[6,7] Finally, thermodynamic corrections and Gibbs free energy profiles were obtained using the Shermo program.[8]

Preparation of NPs.

The nanoparticles were prepared by a modified nanoprecipitation method which was carried out at 25 °C. A mixture of DSPE-PEG (0.5 mg), DSPE-PEG-RGD (0.5 mg)

and 0.5 mg Ir-4 in THF (1 mL) was added into 10 mL of ultrapure water and stirred at room temperature for 8 hours. After dialysis, the nanoparticles Ir-4@R NPs were obtained and stored in a refrigerator at 4 °C for subsequent experiments.

Preparation of Reactive Oxygen and Nitrogen Species (RONS) Solutions.

The RONS solutions containing H_2O_2, TBHP and ClO^-, respectively, were purchased and diluted to the experimental concentration (200 μM) by 1×phosphate buffered saline (PBS) (pH 7.4). In brief, •OH was produced by addition of ferrous chloride (0.1 M, 1 mL) into H_2O_2 solution (1.0 M, 1 mL) through a Fenton reaction. Accordingly, the concentration of •OH is the same as that of Fe^{2+} (50 mM). $O_2^{\bullet -}$ was generated from KO_2 (35.5 mg), which was directly added into dimethyl sulfoxide (10 mL) at a final concentration of 50 mM. $ONOO^-$ was prepared by addition of sodium hydroxide (1.5 M) into mixtures of sodium nitrite (0.6 M), hydrogen peroxide (0.7 M) and hydrochloric acid (0.6 M) at 0 °C, followed by purification through a short column of manganese dioxide to remove excess hydrogen peroxide. The concentration of $ONOO^-$ was determined by measurement of the absorption at 302 nm: $c[ONOO^-] = Abs_{302\ nm}/1.67$ (mM). $^1O_2$ was produced by addition of ClO^- solution (100 mM, 1 mL) into H_2O_2 solution (200 mM, 1 mL). According to this reaction, the concentration of $^1O_2$ is the same as that of ClO^- (50 mM). NO_2^- was generated from $NaNO_2$ (35 mg), which was directly added into deionized water (10 mL) at a final concentration of 50 mM.

Afterglow Luminescence and Fluorescence Imaging In Vitro.

For in vitro afterglow luminescence imaging, including determinations of RONS selectivity, afterglow luminescence spectra, and $ONOO^-$ activated sensitivity, etc., the concentration of Ir-4@R NPs was 100 μg/mL. The afterglow luminescence imaging was carried out with an Xenogen IVIS Lumina II system in a bioluminescent mode (exposure time for 60 s) post RONS (200 μM) addition, unless otherwise specified. Tests were every 20 nm/step by the different filters. The environmental temperature for in vitro afterglow imaging was kept at 37 °C. The fluorescence spectra of Ir-4@R NPs were measured by the same IVIS instrument in a fluorescent mode (exposure time for 1 s). The fluorescence and afterglow luminescence images were analyzed by region of interest (ROI) analysis using the Living Image 4.2 Software.

FITC-Labeled Fibrin Clots Assay.

Fluorescein isothiocyanate (FITC)-labeled fibrin clots were induced by the addition of 10 U mL^{-1} thrombin and 2.5 mM $CaCl_2$ into a fibrinogen solution containing 1 mg mL^{-1} fibrinogen (200 μL) and 1 mg mL^{-1} FITC-labeled fibrinogen (20 μL), followed by incubation at 37 °C for 1 h. The clot was incubated with Ir-4@R NPs (100 μg mL^{-1}, 500 μL) for 1 h and further irradiated with ultrasound (1.0 W cm^{-2}, 1 MHz, 50% duty cycle) and evaluated through a CLSM.

In Vitro Hemolysis Experiments.

A hemolysis assay was conducted to evaluate the hemocompatibility of Ir-4@R NPs.

Firstly, the whole blood of a mouse treated with citrate was gathered and centrifuged at 3000 rpm for 3 min to obtain erythrocytes. Then, the erythrocytes were resuspended in an equal volume of PBS. PBS or water were used as negative and positive controls, respectively, which were added to 2 mL microtubes containing erythrocytes. Various concentrations (5, 10, 50, 100, 150 and 200 μg mL^{-1}) of Ir-4@R NPs were incorporated into the erythrocytes for comparison with the controls. The mixture was then incubated in a water bath at 37 ºC for 2 h, followed by centrifugation at 3000 rpm for 10 min. After centrifugation, 100 μL of supernatant from each sample was transferred to a 96-well plate and the absorbance was recorded with an enzyme marker at 540 nm. The equation for calculating the hemolysis rate (%) is presented below. Hemolysis rate (%) = $(A_0 - A_1) / (A_2 - A_1) \times 100\%$.

In Vitro Thrombus Targeting of Ir-4@R NPs.

Fresh mouse blood was collected and divided into tubes with equal volumes (5 mL). Each tube was mixed with thrombin (5 U μL^{-1}) and $CaCl_2$ (3 mM) to induce the formation of clots and cut into equal sizes. The artificial thrombus was then incubated with PBS, Ir-4 NPs [no c(RGDfC)] or Ir-4@R NPs aqueous solution (100 μg mL^{-1}) to verify the targeting ability of the NPs. After incubation for 1 h, 3 h or 5 h, the thrombus clots were taken out. The chemiluminescence of the clots was analyzed using an IVIS imaging system.

Construction of mice carotid artery thrombus model.

Male C57 BL/6J mice (8-10 weeks old) were anesthetized using isoflurane (2% of isoflurane, 0.5 mL min^{-1} oxygen) and the neck hair was shaved off. Then the skin around the neck was cut with surgical scissors and the connective tissue and fat were peeled away to expose the carotid vessels. A 10% aqueous solution of $FeCl_3$-soaked filter paper (2 × 1 mm) was placed on the surface of the exposed carotid artery for 10 min, then the filter paper was removed. The vessel and surrounding tissue were washed with sterilized saline solution. An apparent aggregated embolus could be observed under a somatic microscope. A laser speckle imaging system (RWD, RFLSI III, Shenzhen, China) was used to monitor the hemodynamic changes before and after the induction of a carotid artery thrombus using $FeCl_3$. All animal procedures were approved by the China Technology Industry Holdings (Shenzhen) Co., Ltd.

In Vivo Afterglow Luminescence Imaging of Thrombus.

The C57 BL/6J mice (8-10 weeks old) with a thrombus model or normal C57 BL/6J mice (8-10 weeks old) were intravenously administrated with Ir-4@R NPs (5 mg/kg). The mice were then anesthetized using isoflurane (2% of isoflurane, 0.5 mL min^{-1} oxygen), and placed on a heating platform at 37 °C. At predetermined times (0, 15, 30, 45, 60 and 75 min) post NPs administration, in vivo afterglow luminescence imaging was carried out using an IVIS imaging system.

Evaluation of the Thrombolytic Effect of Different Formulations In Vivo.

To evaluate the therapeutic effect of different formulations on the thrombi, the mice

with carotid thrombosis or lower extremity arterial thrombosis were randomly divided into three groups (n = 3): PBS, free UK, and Ir-4@R NPs. The different formulations were intravenously injected into the mouse via the tail vein. At different times (0, 10, 20, 30, 60 and 120 min) after the treatments, a laser speckle imaging system was used to observe and record the blood flow velocity and blood flow at the thrombosis sites, and applied to measure the blood flow velocities. Finally, the mouse was euthanized, and the carotid arteries were excised for section and H&E staining. The areas and morphology of the thrombi after different treatments were observed under an optical microscope. The sections were analyzed using the Image J2x software (version 2.1.4.7) and thrombolytic efficiency was determined by the area ratio of vascular occlusion to total vascular lumen.

In Vivo Biosafety Evaluation.

To test the potential toxicity of Ir-4@R NPs, healthy male C57 BL/6J mice (8-10 weeks old) were intravenously injected with PBS, free UK, or Ir-4@R NPs (5 mg/kg). One week after PBS, free UK or NPs administration, the mice were euthanized and blood was withdrawn. Then the hematological parameters were analyzed using an automated hematology analyzer. The major organs (brain, heart, liver, spleen, lung, and kidneys) were also harvested for histological analysis. The tissues were fixed in 4% paraformaldehyde (PFA), followed by embedding in paraffin and sectioning at a thickness of 5 μm for H&E staining. The slices were observed under a digital microscope (Leica QWin).

Animal Experiments.

All animal experiments were approved by the Ethics Committee for Animal Experimentation of China Technology Industry Holdings (Shenzhen) Co., Ltd (Ethics approval number: 20240030). All procedures complied with the Animal Research: Reporting In Vivo Experiments (ARRIVE) guidelines. The mice were housed in a pathogen-free environment with no more than three mice per cage.

Synthesis.

Complex Ir-1 (Scheme S1): $[Ir(ppy)_2Cl]_2$ (0.102 g, 0.1 mmol) and auxiliary ligand L1 (0.029 g, 0.2 mmol) were dissolved in a mixture of methanol (30 mL) and dichloromethane (30 mL) and refluxed for 6 h under the protection of N_2. After cooling to room temperature, excess KPF_6 was added to the mixture (to replace counterion Cl^-). After stirring for 0.5 h, the mixture was filtered to remove the excess potassium salts, and the solvent was removed from the filtrate by rotary evaporation to obtain the crude product. Finally, the purple solid product was purified by column chromatography on silica gel (eluent dichloromethane /ethyl acetate 10/1, v/v) to give Ir-1 with a yield of 60%. ^{1}H NMR (500 MHz, $CDCl_3$, δ) 8.43 (d, J = 8.0 Hz, 1H), 7.84 (t, J = 7.6 Hz, 2H), 7.76 (t, J = 7.7 Hz, 1H), 7.69 – 7.65 (m, 4H), 7.64 – 7.61 (m, 3H), 7.27 (s, 1H), 6.98 (dd, J = 12.7, 5.6 Hz, 2H), 6.93 (t, J = 7.7 Hz, 2H), 6.89 (dd, J = 12.4, 5.3 Hz, 2H), 6.82 (t, J = 7.3 Hz, 1H), 6.53 (s, 1H), 6.44 (d, J = 7.4 Hz, 1H), 6.34

(d, J = 7.5 Hz, 1H). HRMS (ESI) *m*/*z*: $[M + H]^+$ calcd for $C_{30}H_{23}IrN_5$,646.16; found, 646.1636.

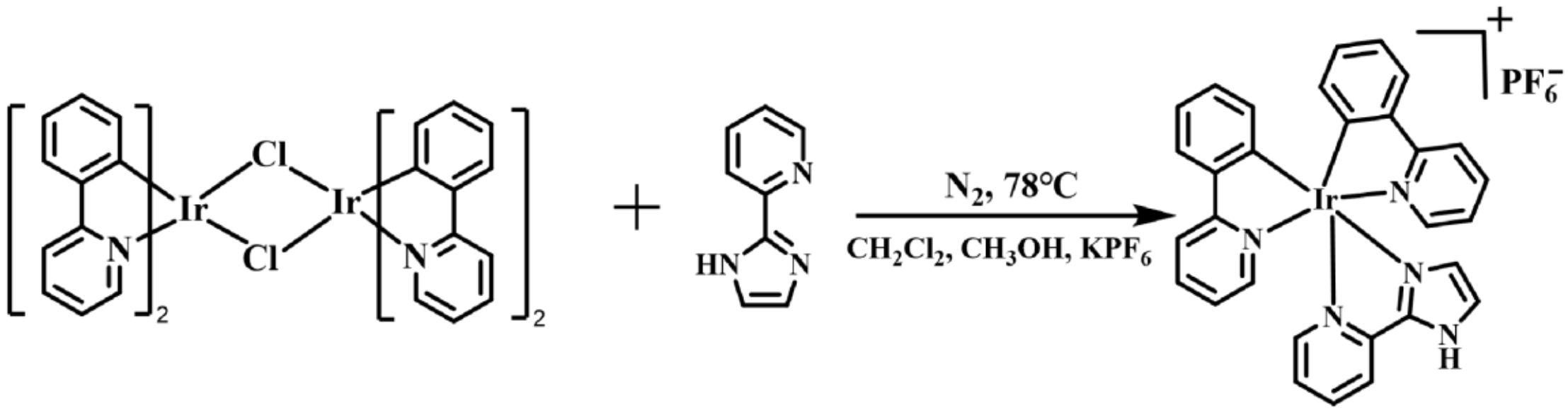

Scheme S1. Synthetic route to Ir-1.

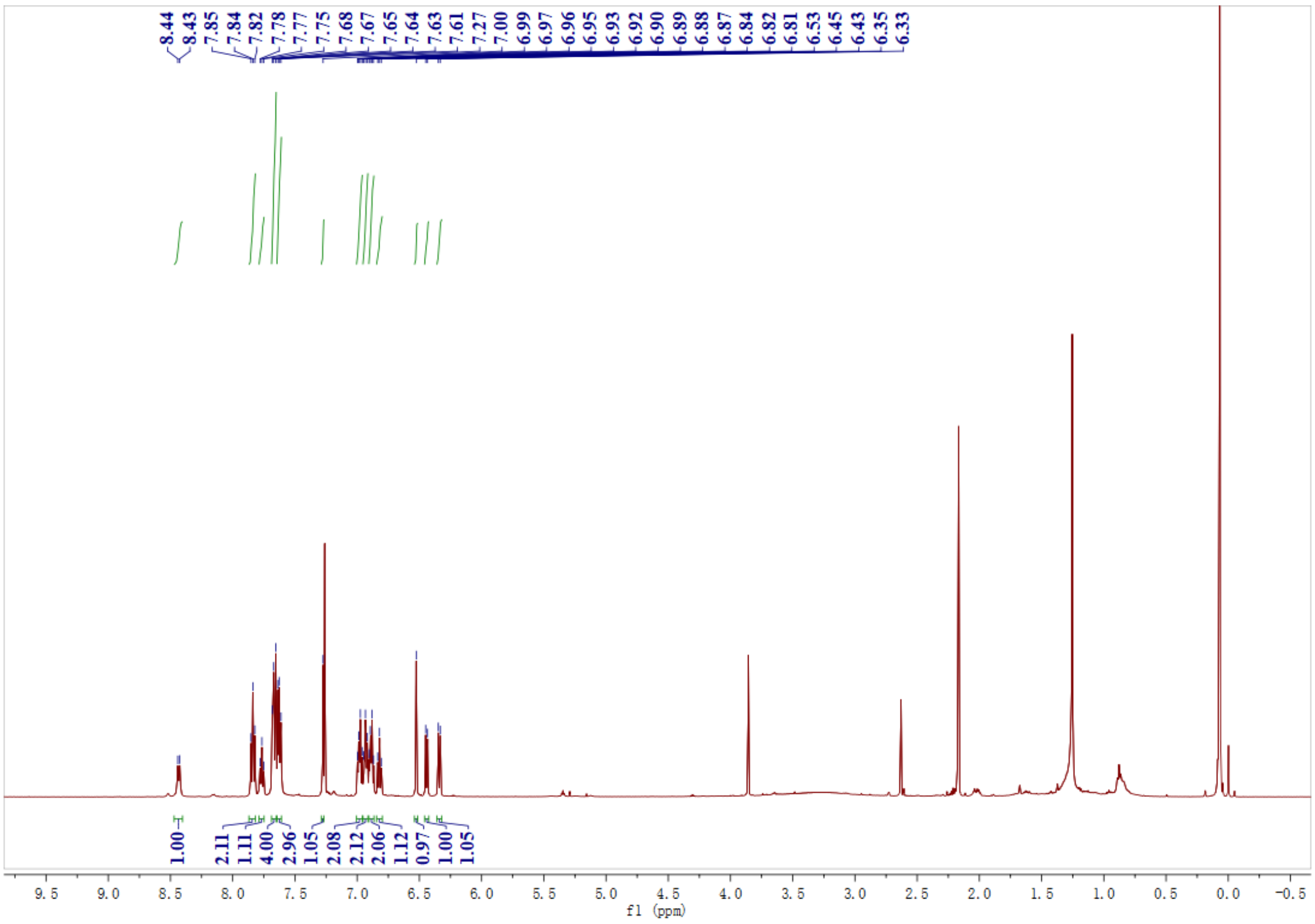

Figure S1. ^1H NMR spectrum of Ir-1 in $CDCl_3$ at room temperature.

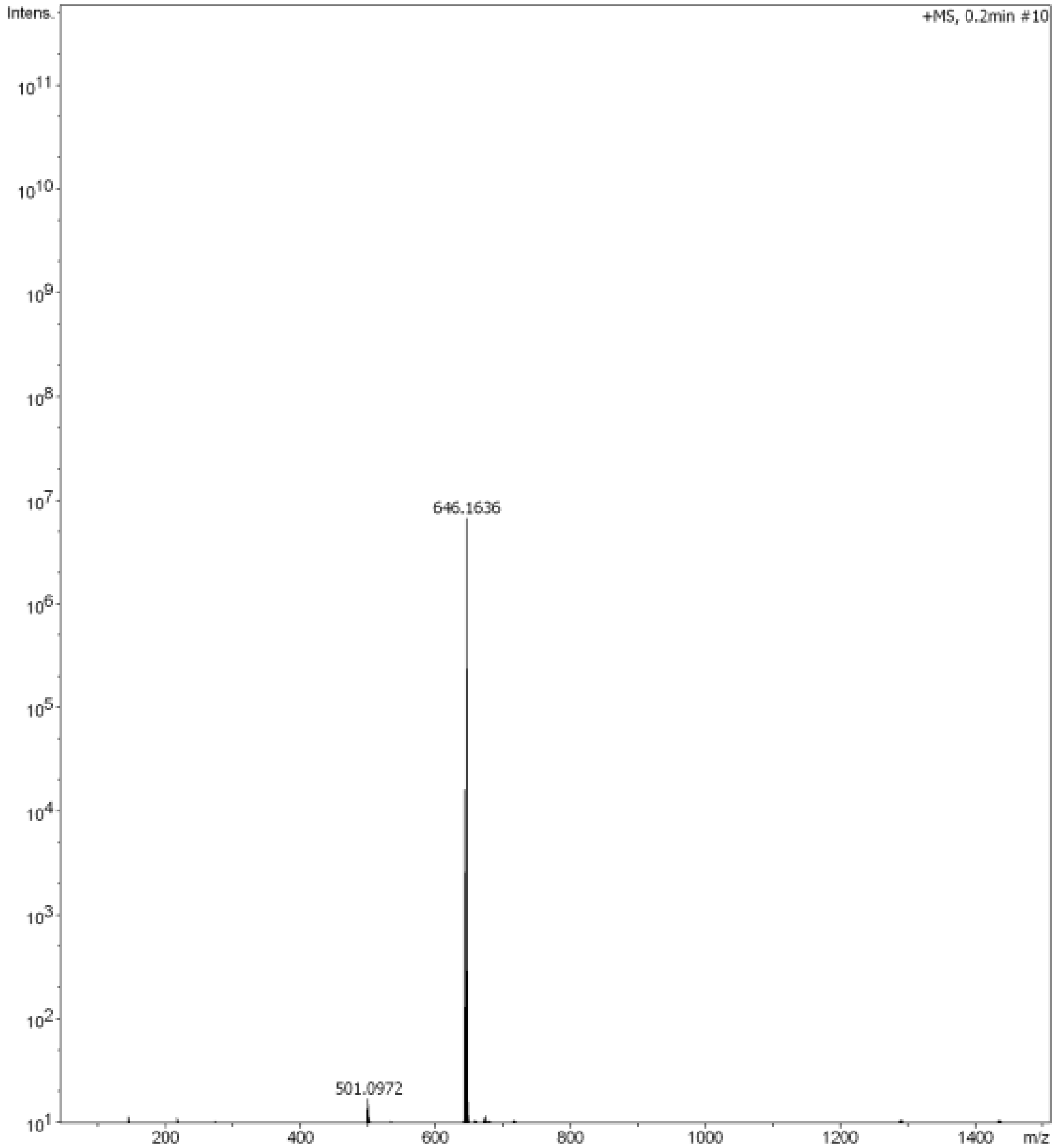

Figure S2. ESI mass spectrum of Ir-1 at room temperature.

Complex Ir-2 (Scheme S2): $[Ir(ppy)_2Cl]_2$ (0.102 g, 0.1 mmol) and auxiliary ligand L2 (0.039 g, 0.2 mmol) were dissolved in a mixture of methanol (30 mL) and dichloromethane (30 mL) and the mixture was refluxed for 6 h under the protection of N_2. After cooling to room temperature, excess KPF_6 was added to the mixture (to replace counterion Cl^-). Work-up as described above for Ir-1 gave Ir-2 with a yield of 80%. ^{1}H NMR (600 MHz, $CDCl_3$ δ) 8.67 (d, J = 8.0 Hz, 1H), 8.09 (t, J = 7.8 Hz, 1H), 7.91 (d, J = 7.6 Hz, 2H), 7.85 (dd, J = 16.5, 8.4 Hz, 2H), 7.74 (t, J = 7.8 Hz, 1H), 7.69 (d, J = 7.0 Hz, 3H), 7.66 (d, J = 5.7 Hz, 1H), 7.47 (d, J = 5.6 Hz, 1H), 7.38 (t, J = 6.4 Hz, 1H), 7.34 (t, J = 7.8 Hz, 1H), 7.10 (t, J = 7.5 Hz, 1H), 7.06 (t, J = 7.6 Hz, 1H), 7.00 (t, J = 7.8 Hz, 1H), 6.98 – 6.92 (m, 4H), 6.43 (d, J = 7.6 Hz, 1H), 6.37 (d, J = 7.5 Hz, 1H), 6.23 (d, J = 8.5 Hz, 1H). HRMS (ESI) m/z: $[M + H]^+$ calcd for $C_{34}H_{25}IrN_5$, 696.17; found, 696.1759.

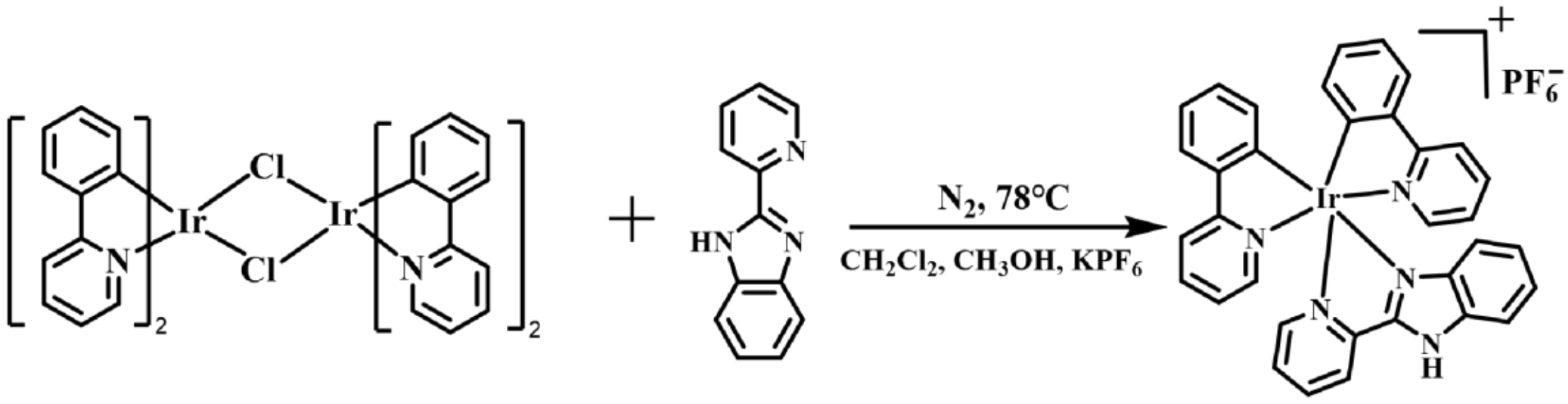

Scheme S2. Synthetic route to Ir-2.

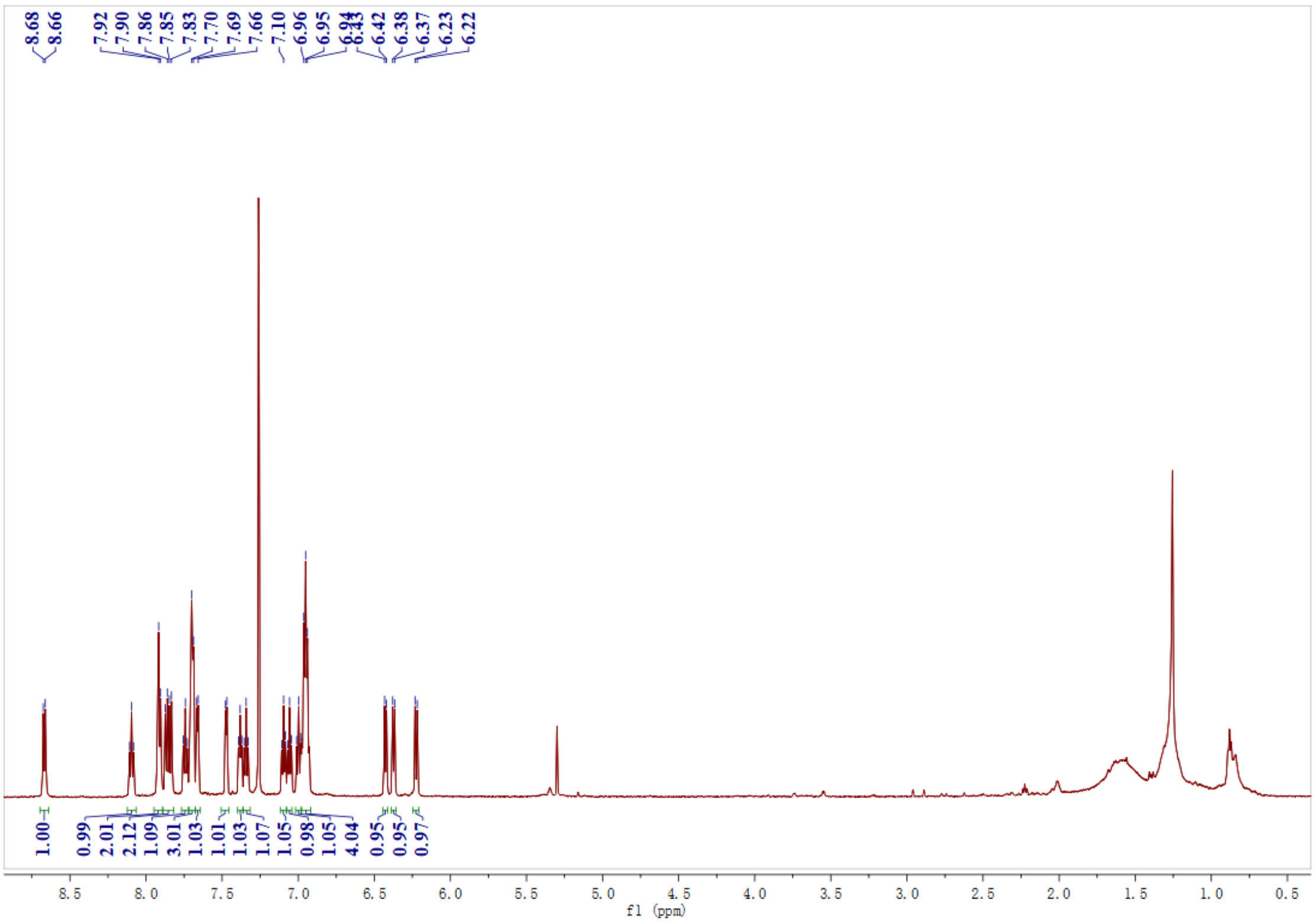

Figure S3. ^1H NMR spectrum of Ir-2 in $CDCl_3$ at room temperature.

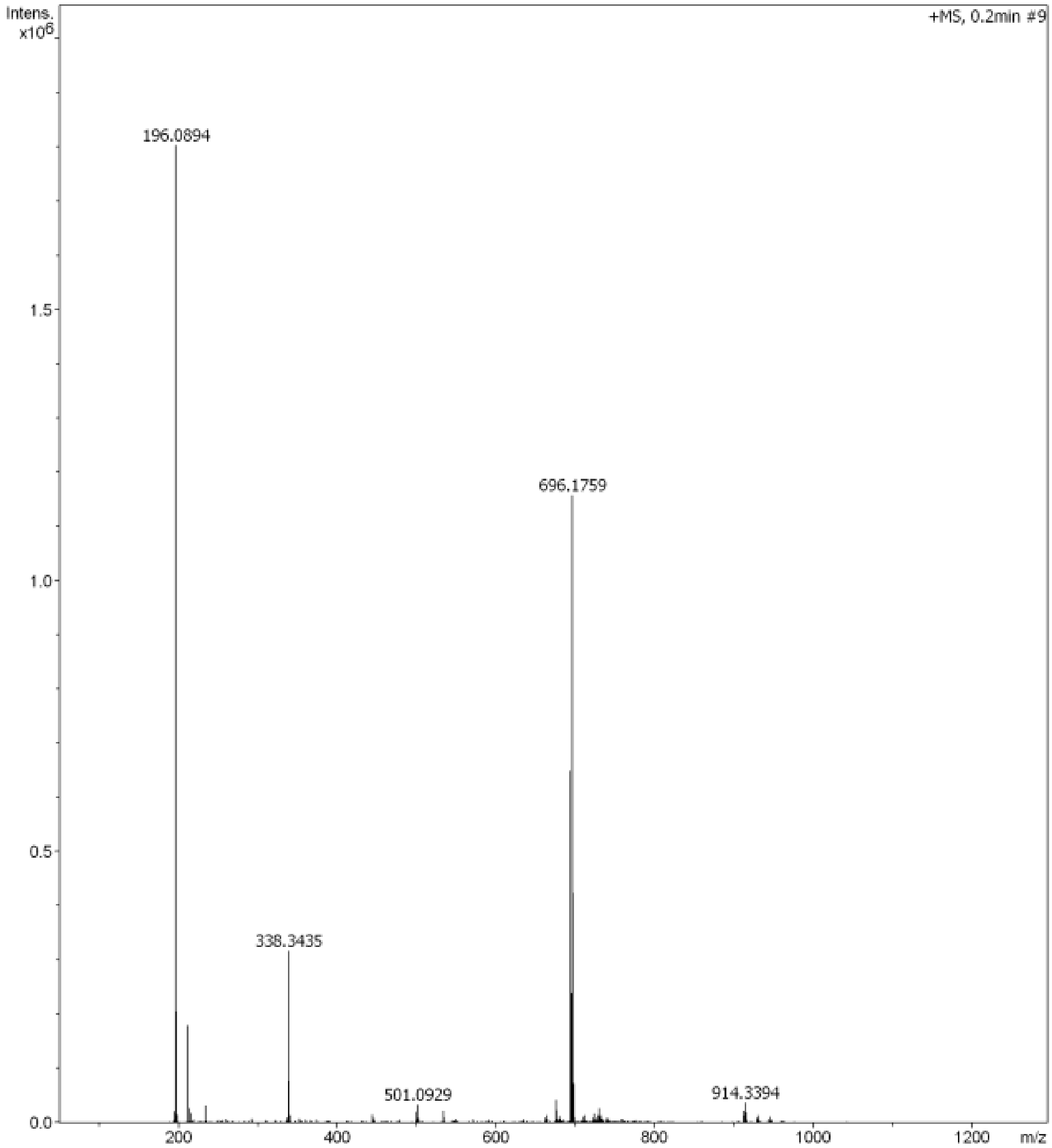

Figure S4. ESI mass spectrum of Ir-2 at room temperature.

Complex Ir-3 (Scheme S3): $[Ir(ppy)_2Cl]_2$ (0.102 g, 0.1 mmol) and auxiliary ligand L3 (0.0442 g, 0.2 mmol) were dissolved in a mixture of methanol (30 mL) and dichloromethane (30 mL) and refluxed for 6 h under the protection of N_2. After cooling to room temperature, excess KPF_6 was added to the mixture (to replace counterion Cl^-). Work-up as described above for Ir-1 gave Ir-3 with a yield of 60%. ^{1}H NMR (600 MHz, DMSO-d_6 δ) 8.21 (t, J = 8.7 Hz, 2H), 7.93 (d, J = 8.0 Hz, 1H), 7.91 (d, J = 8.6 Hz, 1H), 7.88 (d, J = 8.0 Hz, 1H), 7.86 – 7.80 (m, 4H), 7.78 (d, J = 5.4 Hz, 1H), 7.74 – 7.66 (m, 5H), 7.48 – 7.43 (m, 1H), 7.23 (t, J = 6.6 Hz, 1H), 7.18 (t, J = 6.6 Hz, 1H), 6.98 – 6.91 (m, 2H), 6.89 (d, J = 8.3 Hz, 1H), 6.83 (dt, J = 18.2, 7.4 Hz, 2H), 6.59 (s, 1H), 6.21 – 6.16 (m, 2H). HRMS (ESI) m/z: $[M + H]^+$ calcd for $C_{36}H_{27}IrN_5$, 722.19; found, 722.11.

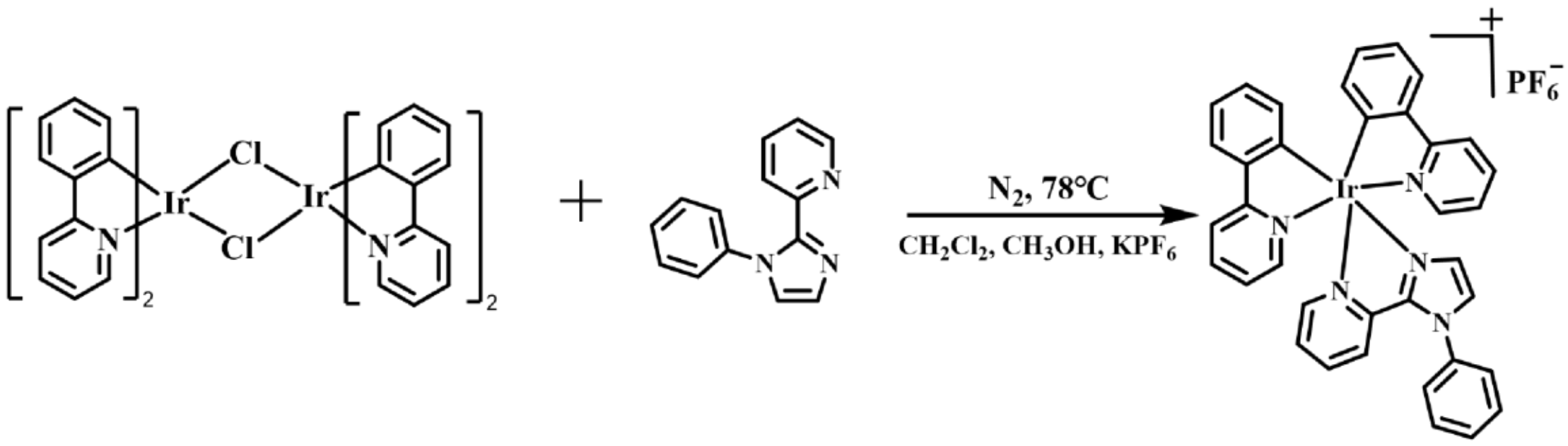

Scheme S3. Synthetic route to Ir-3**.**

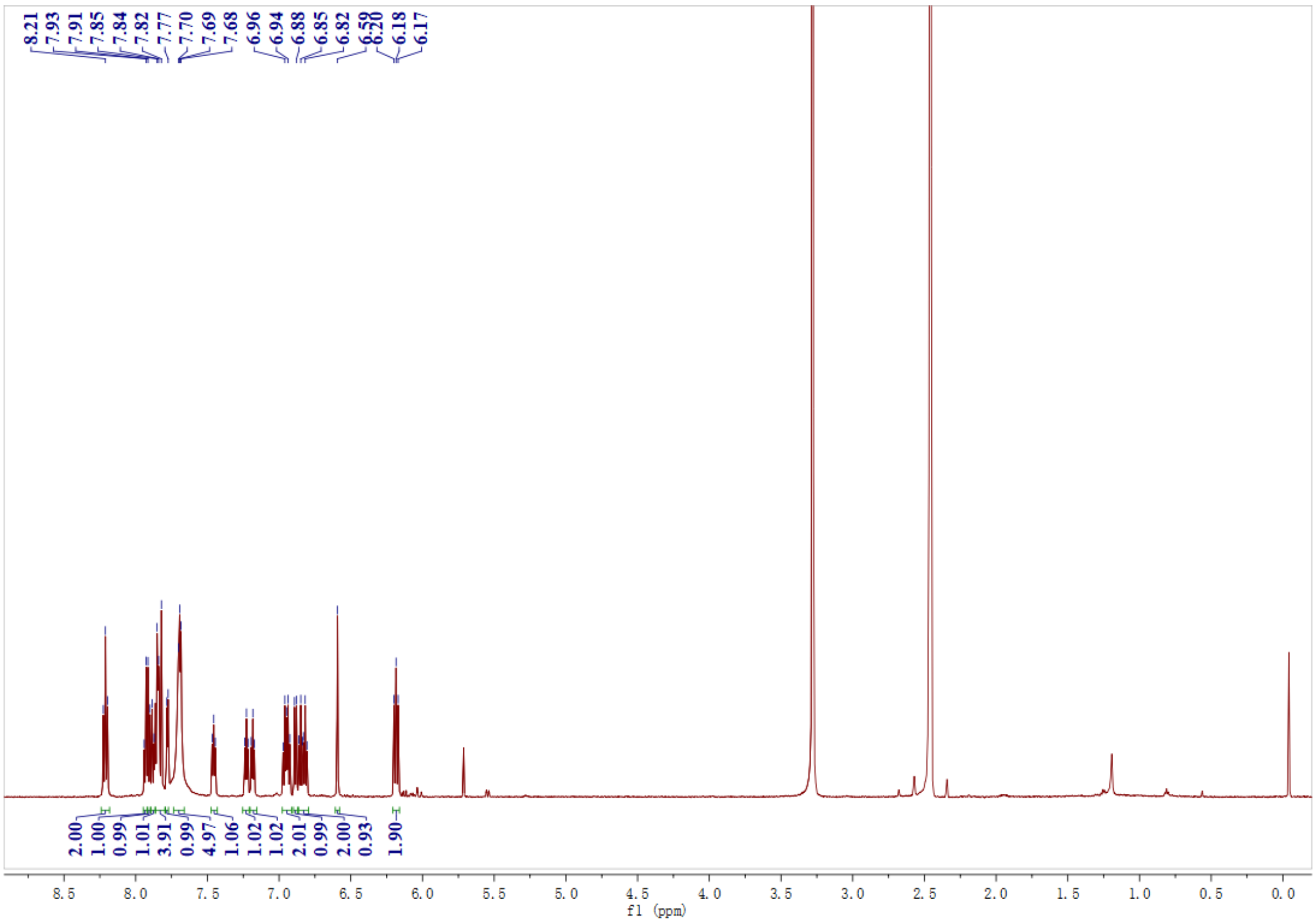

Figure S5. ^{1}H NMR spectrum of Ir-3 in DMSO-d_6 at room temperature.

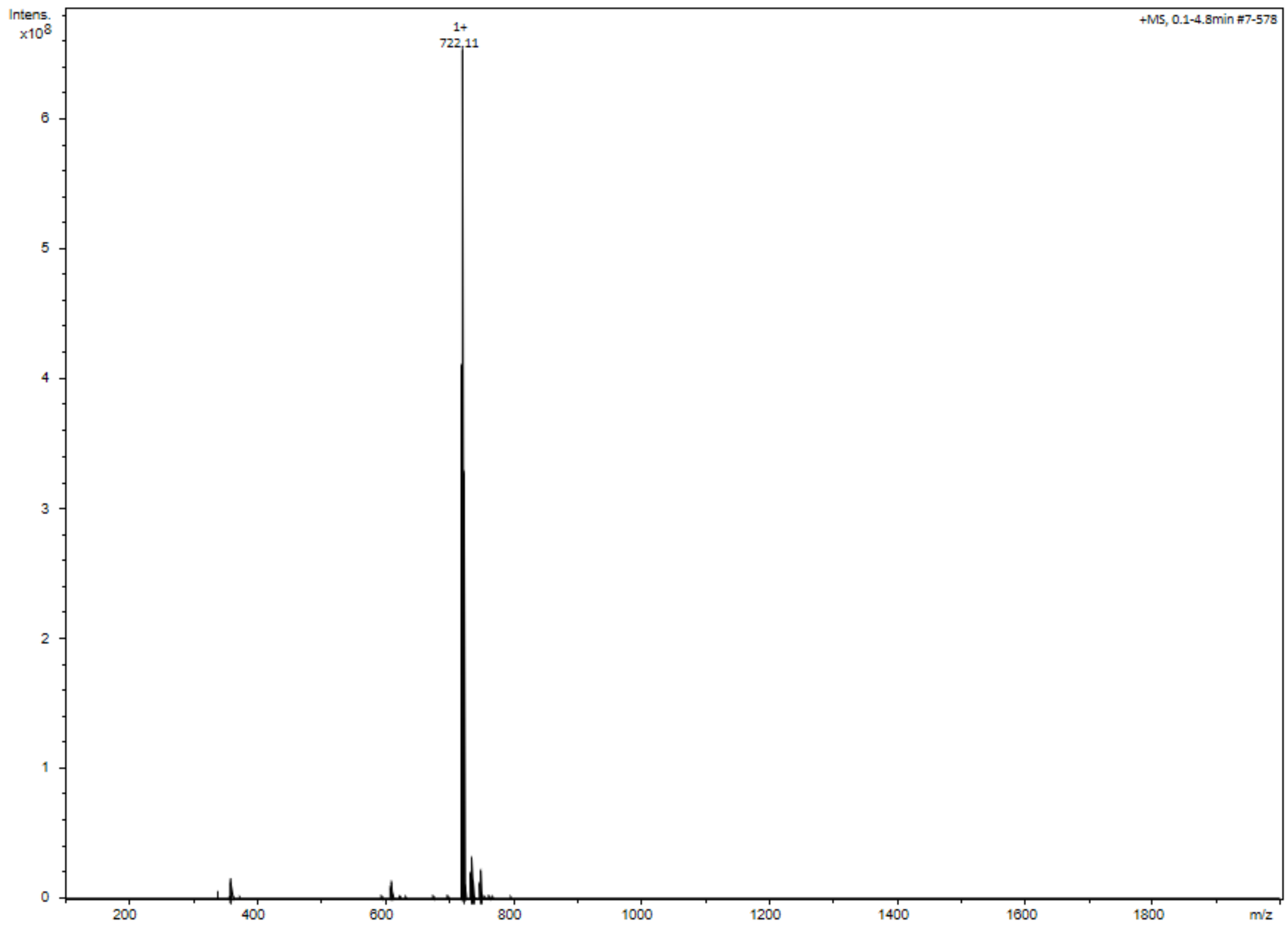

Figure S6. ESI mass spectrum of Ir-3 at room temperature.

Complex Ir-4 (Scheme S4): $[Ir(TPA)_2Cl]_2$ [9] (0.1836 g, 0.1 mmol) and auxiliary ligand L2 (0.039 g, 0.2 mmol) were dissolved in a mixture of methanol (30 mL) and dichloromethane (30 mL) and refluxed for 6 h under the protection of N_2. After cooling to room temperature, excess KPF_6 was added to the mixture (to replace counterion Cl^-). Work-up as described above for Ir-1 gave Ir-4 with a yield of 40%. ^{1}H NMR (600 MHz, DMSO-d_6) δ 8.76 – 8.73 (m, 2H), 8.43 (d, J = 8.1 Hz, 1H), 8.11 (dd, J = 19.4, 9.9 Hz, 3H), 7.80 – 7.77 (m, 1H), 7.74 – 7.71 (m, 4H), 7.68 (d, J = 5.3 Hz, 1H), 7.58 (d, J = 7.7 Hz, 1H), 7.51 (d, J = 6.8 Hz, 2H), 7.17 (d, J = 6.5 Hz, 1H), 7.12 (d, J = 6.4 Hz, 1H), 7.07 (s, 1H), 7.05 (d, J = 7.8 Hz, 3H), 7.02 (d, J = 7.8 Hz, 4H), 7.00 (s, 1H), 6.97 (d, J = 6.5 Hz, 1H), 6.91 (d, J = 8.1 Hz, 4H), 6.89 (d, J = 7.5 Hz, 2H), 6.85 (d, J = 8.2 Hz, 4H), 6.83 (d, J = 7.3 Hz, 2H), 6.71 (dd, J = 8.8, 2.3 Hz, 1H), 6.61 (dd, J = 8.9, 2.4 Hz, 1H), 6.21 – 6.13 (m, 2H), 6.06 (dd, J = 17.2, 2.0 Hz, 1H), 5.84 (d, J = 2.2 Hz, 1H), 5.79 (d, J = 2.1 Hz, 1H), 5.59 (dd, J = 10.2, 1.7 Hz, 1H). HRMS (ESI) m/z: $[M + H]^+$ calcd for $C_{66}H_{47}IrN_7$, 1130.35; found, 1130.3561.

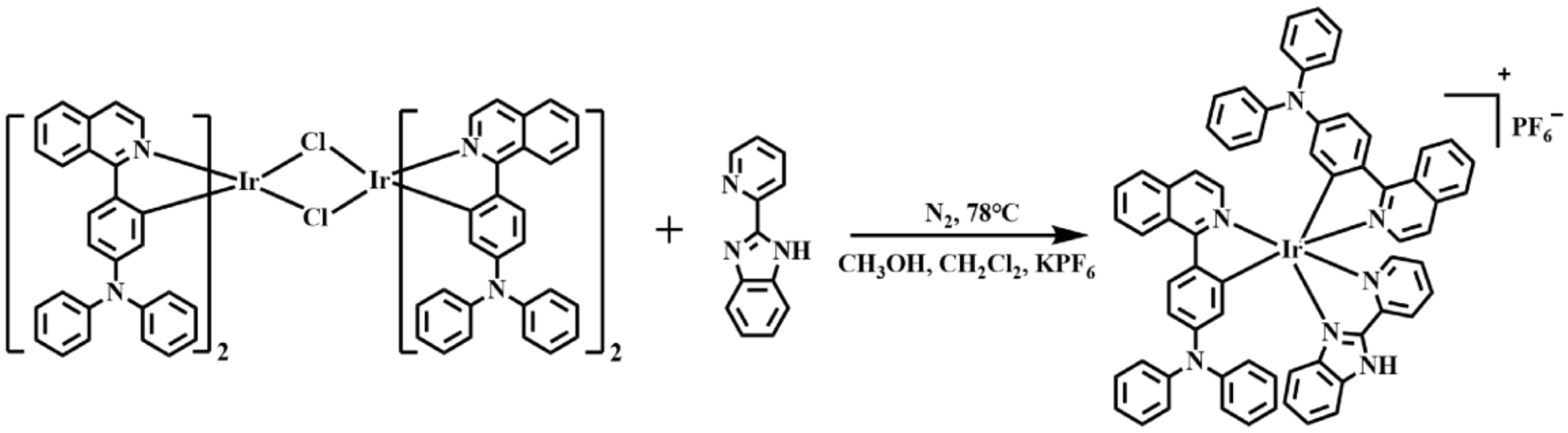

Scheme S4. Synthetic route to Ir-4**.**

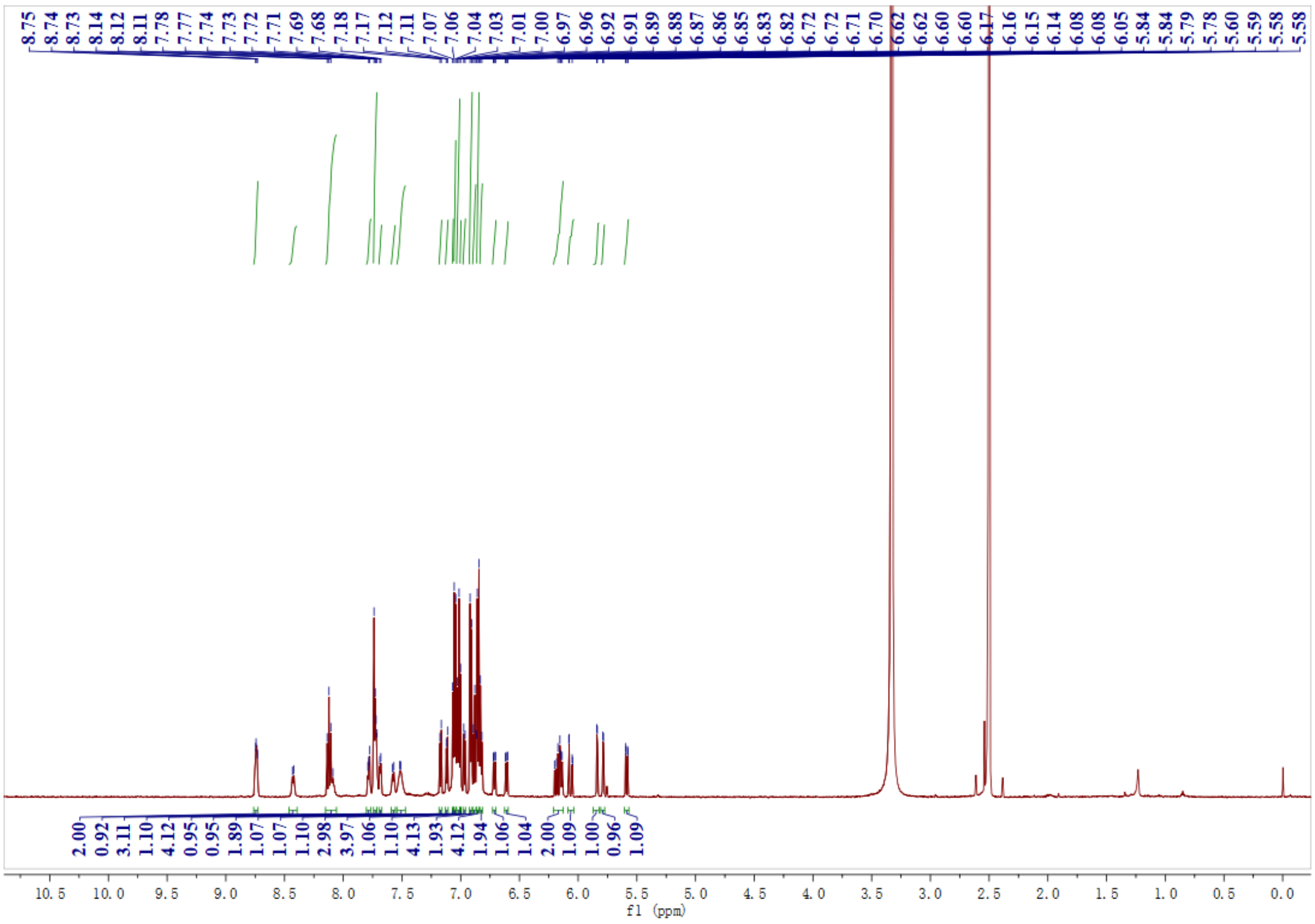

Figure S7. ^{1}H NMR spectrum of Ir-4 in DMSO-d_6 at room temperature.

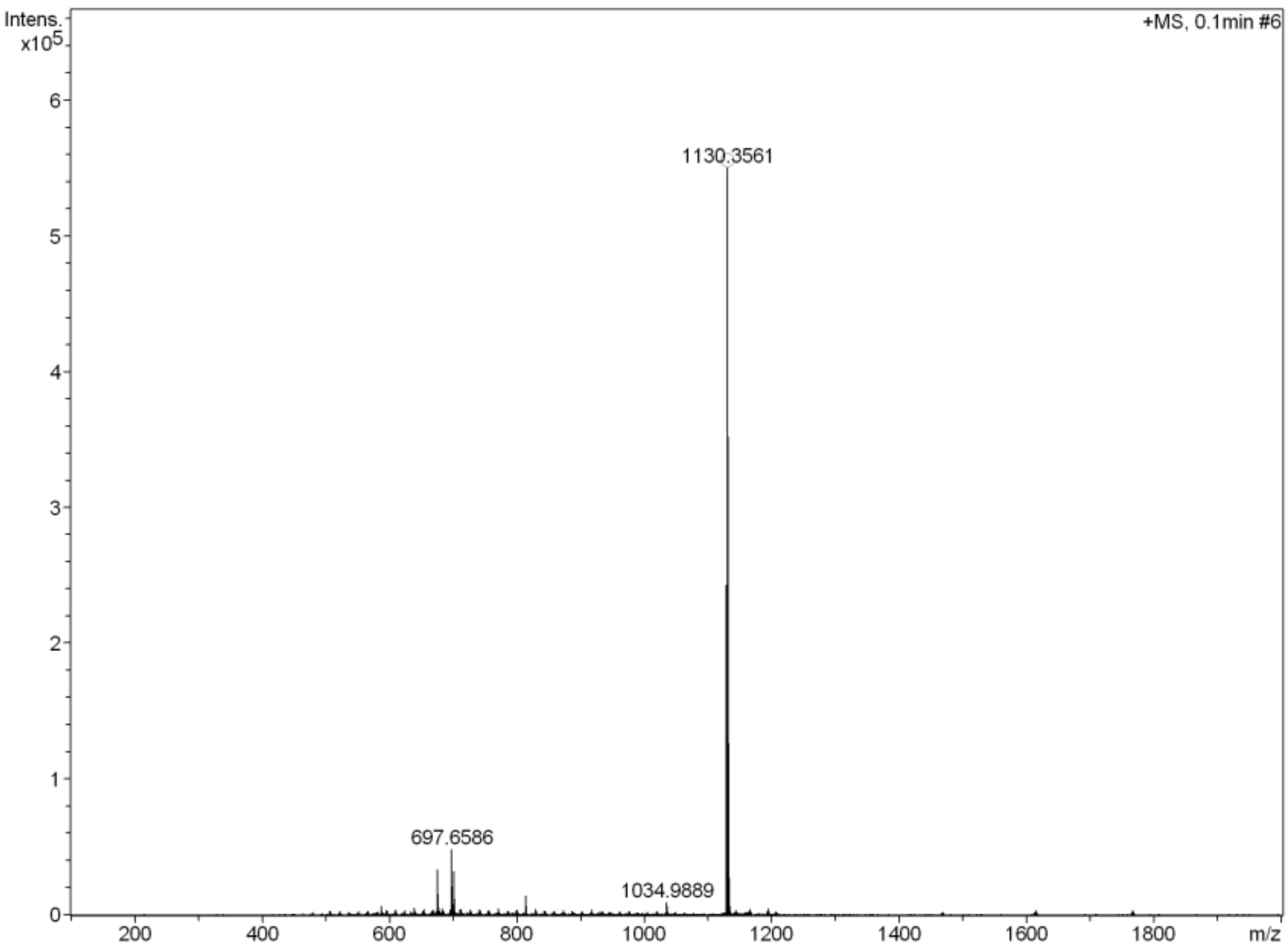

Figure S8. ESI mass spectrum of Ir-4 at room temperature.

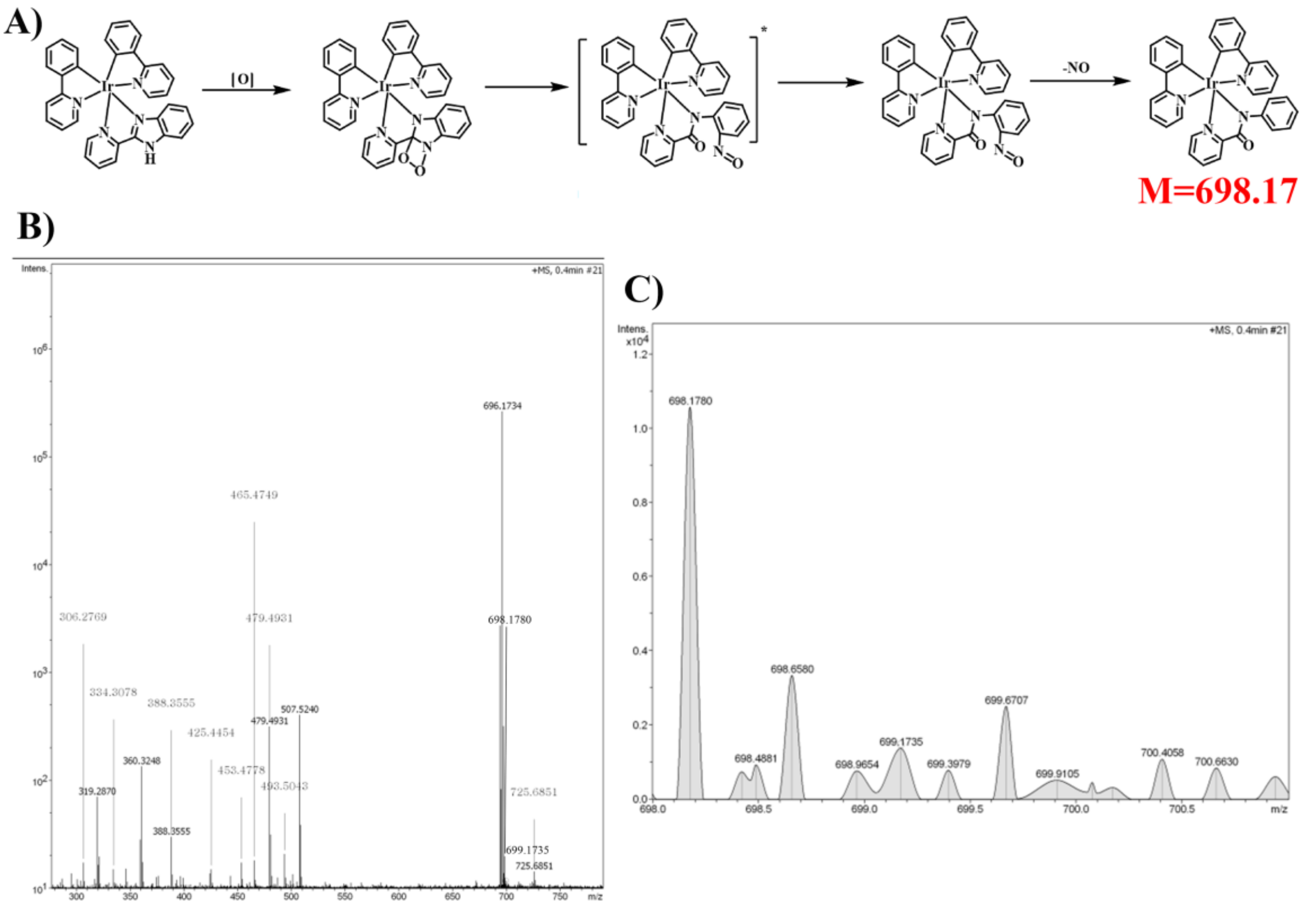

Figure S9. A) Proposed mechanism for the generation of afterglow luminescence ([O] = $ONOO^{-}$); B) ESI mass spectrum of afterglow luminescent products of Ir-2 at room temperature; C) Local area ESI mass spectrum corresponding to B).

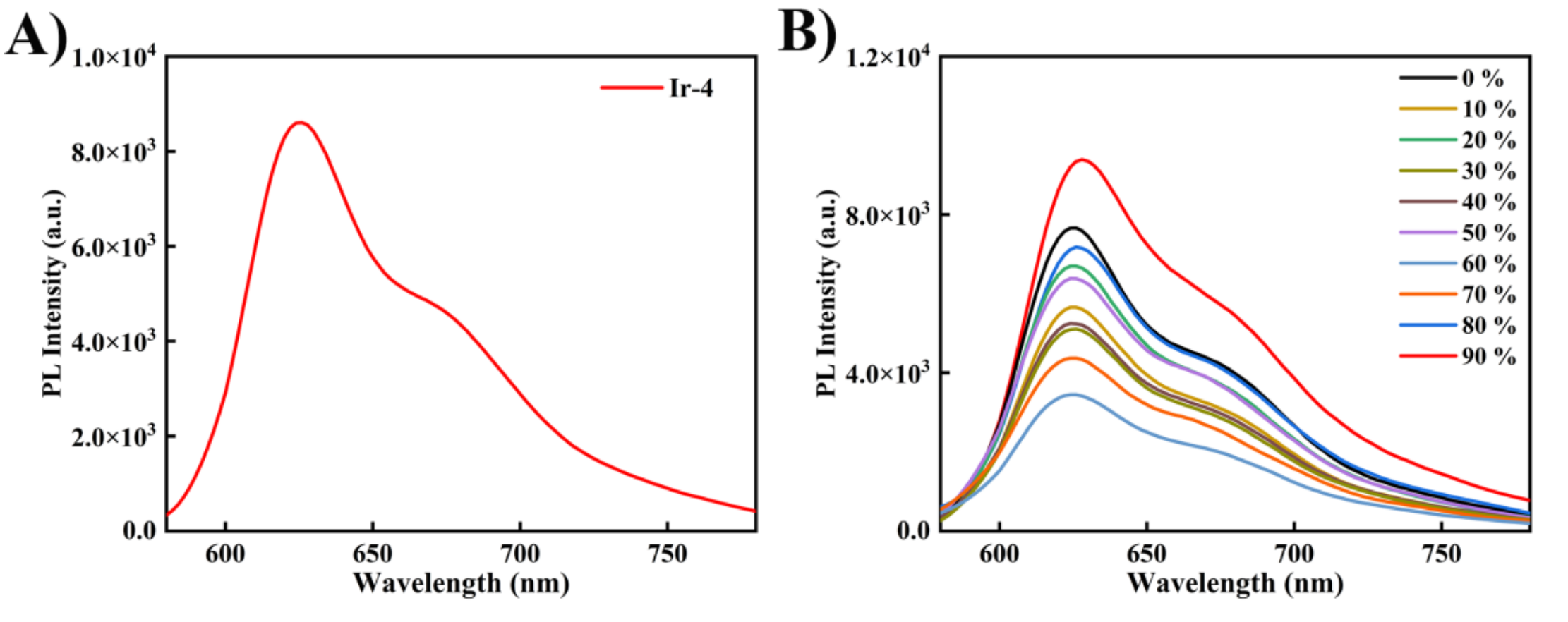

Figure S10. A) Fluorescence spectra of Ir-4 in CH_3OH (complex concentration = 1.0 $\times 10^{-5}$ M); C) Emission spectra of Ir-4 in CH_3OH-H_2O mixtures (complex concentration = 1.0 × 10^{-5} M) with different water fractions (0%–90% v/v) at room temperature.

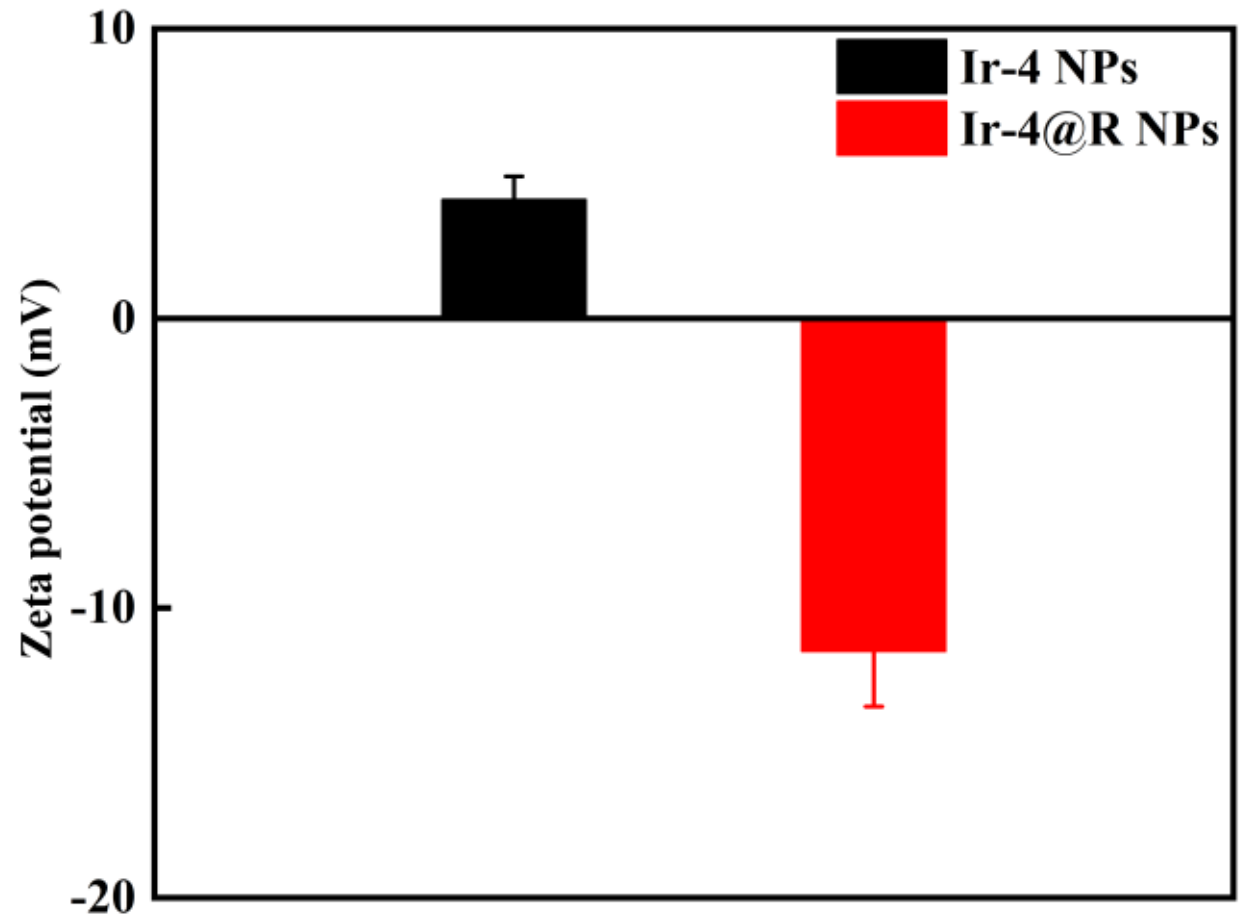

Figure S11. Zeta potential values of Ir-4 NPs and Ir-4@R NPs in water.

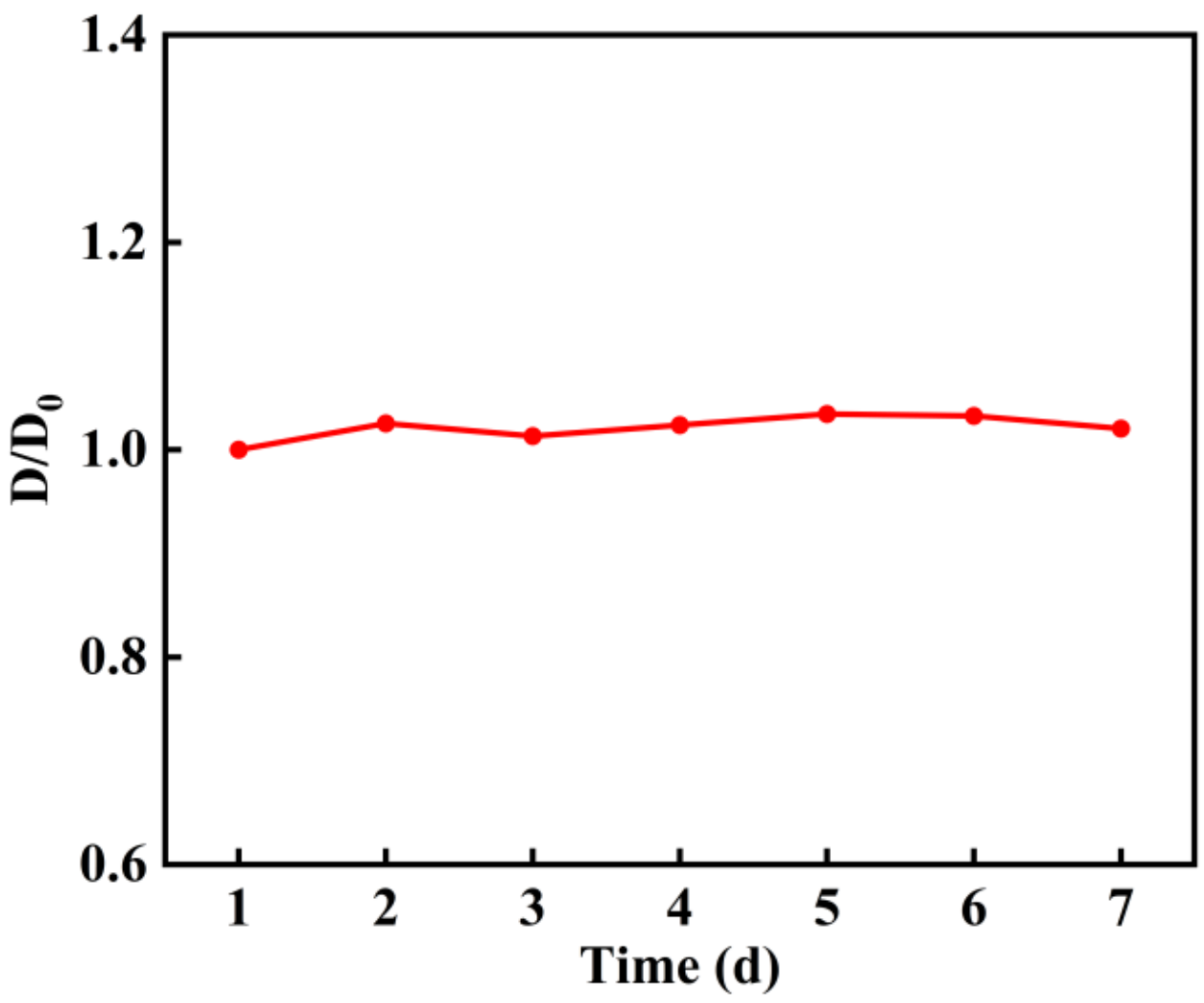

Figure S12. Stability analysis of Ir-4@R NPs at room temperature in H_2O measured by DLS.

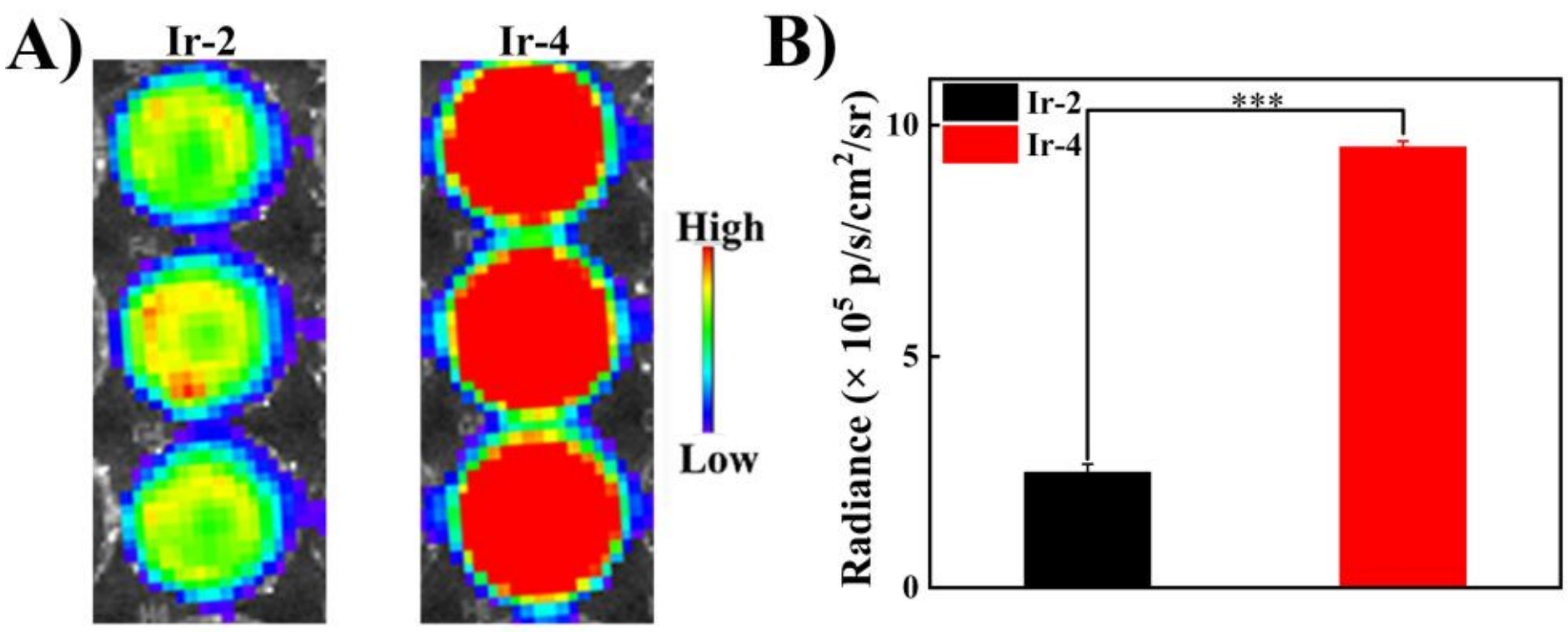

Figure S13. A) The afterglow luminescence images of the mixture of Ir-2 or Ir-4 and ONOO⁻ (100 μM) in $CH_3OH:H_2O$ = 1:1 v/v solution acquired by IVIS imaging system; B) The quantification of corresponding afterglow luminescence intensities in (A).

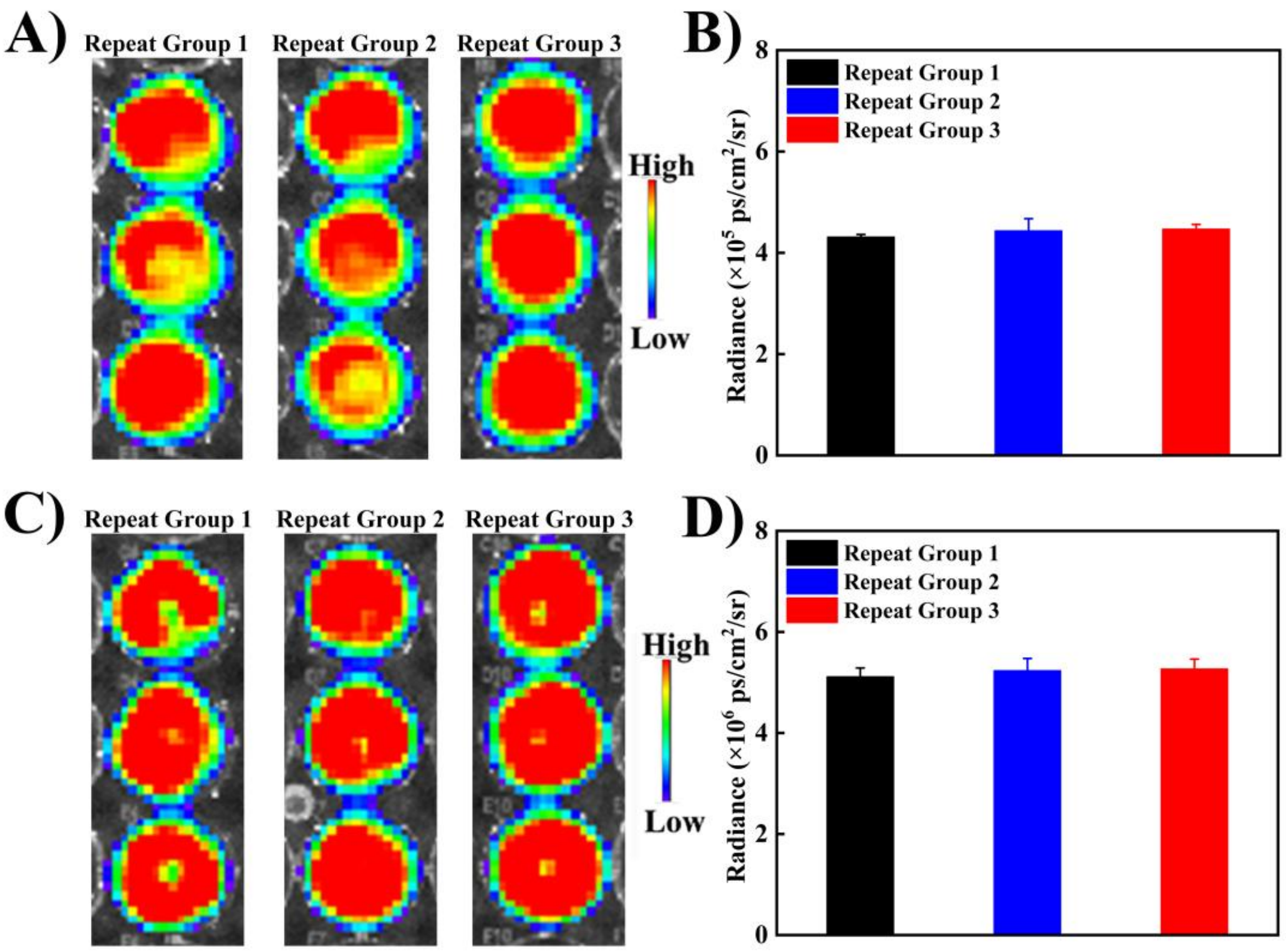

Figure S14. A) The afterglow luminescence images of three separate repeat batches of Ir-4@R NPs under US pre-irradiation for three minutes (1.0 W cm^{-2}, 1 MHz, 50% duty cycle) acquired by IVIS imaging system; B) The quantification of the corresponding afterglow luminescence intensities in (A); C) The afterglow luminescence images after the mixture of Ir-4@R NPs and $ONOO^-$ (100 μM) acquired by IVIS imaging system; D) The quantification of corresponding afterglow luminescence intensities in (C).

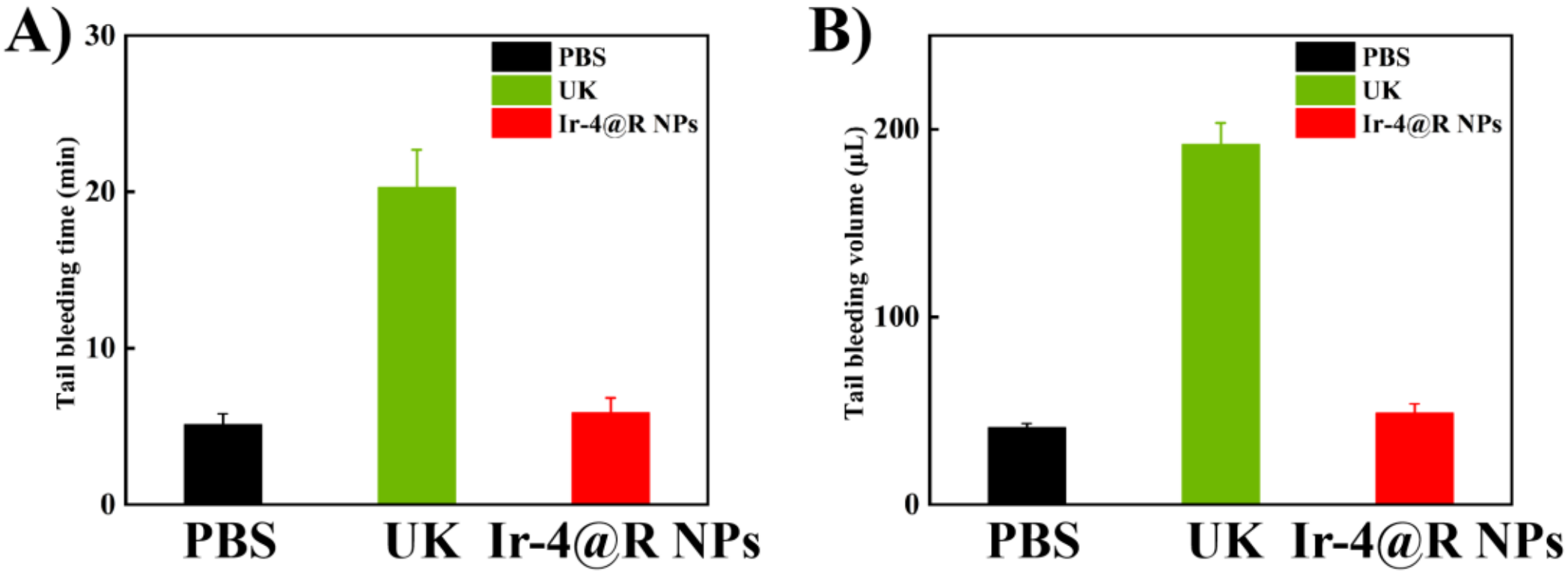

Figure S15. A) The tail bleeding time of mice after different treatments. Data are presented as mean ± SD (n = 3); B) The tail bleeding volume of mice after different treatments. Data are presented as mean ± SD (n = 3).

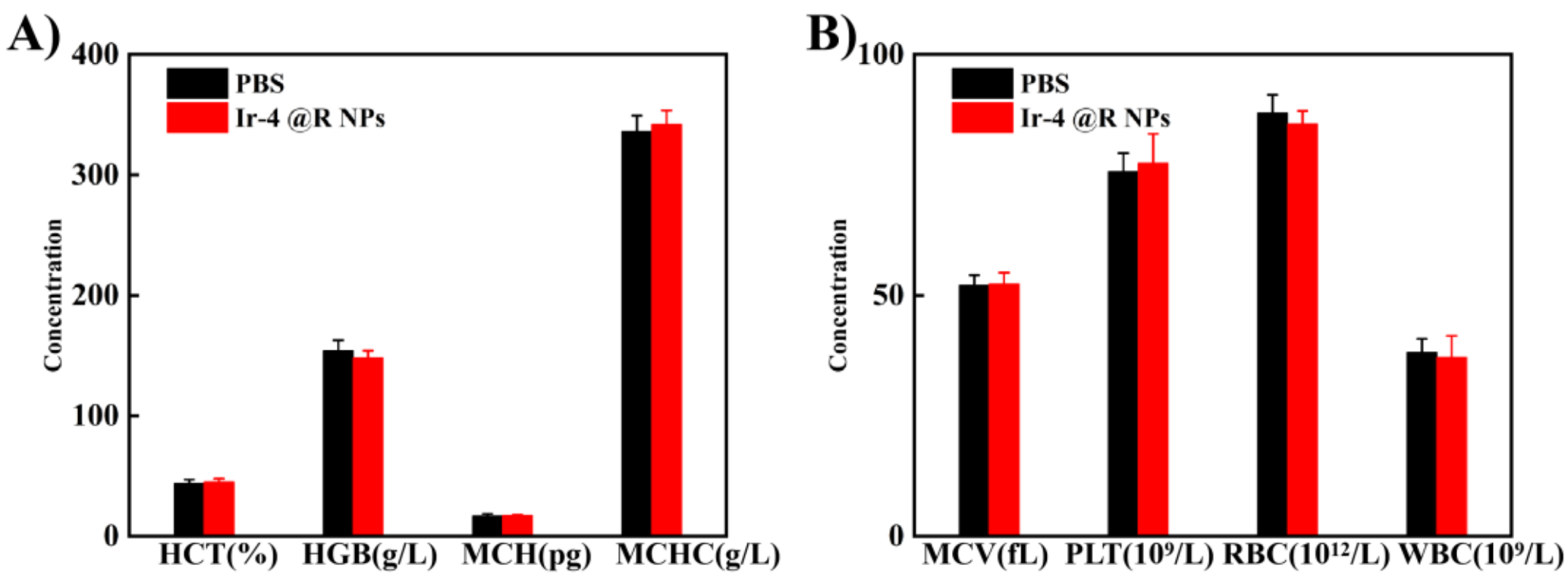

Figure S16. Blood test parameters for HCT, HGB, MCH, MCHC, MCV, PLT, RBC and WBC count of mice with different treatments. HCT is Hematocrit; HGB is Hemoglobin; MCH is Mean Corpuscular Hemoglobin; MCHC is Mean Corpuscular Hemoglobin Concentration; MCV is Mean Corpuscular Volume; PLT is Platelet Count; RBC is Red Blood Cells; WBC is White Blood Cells.

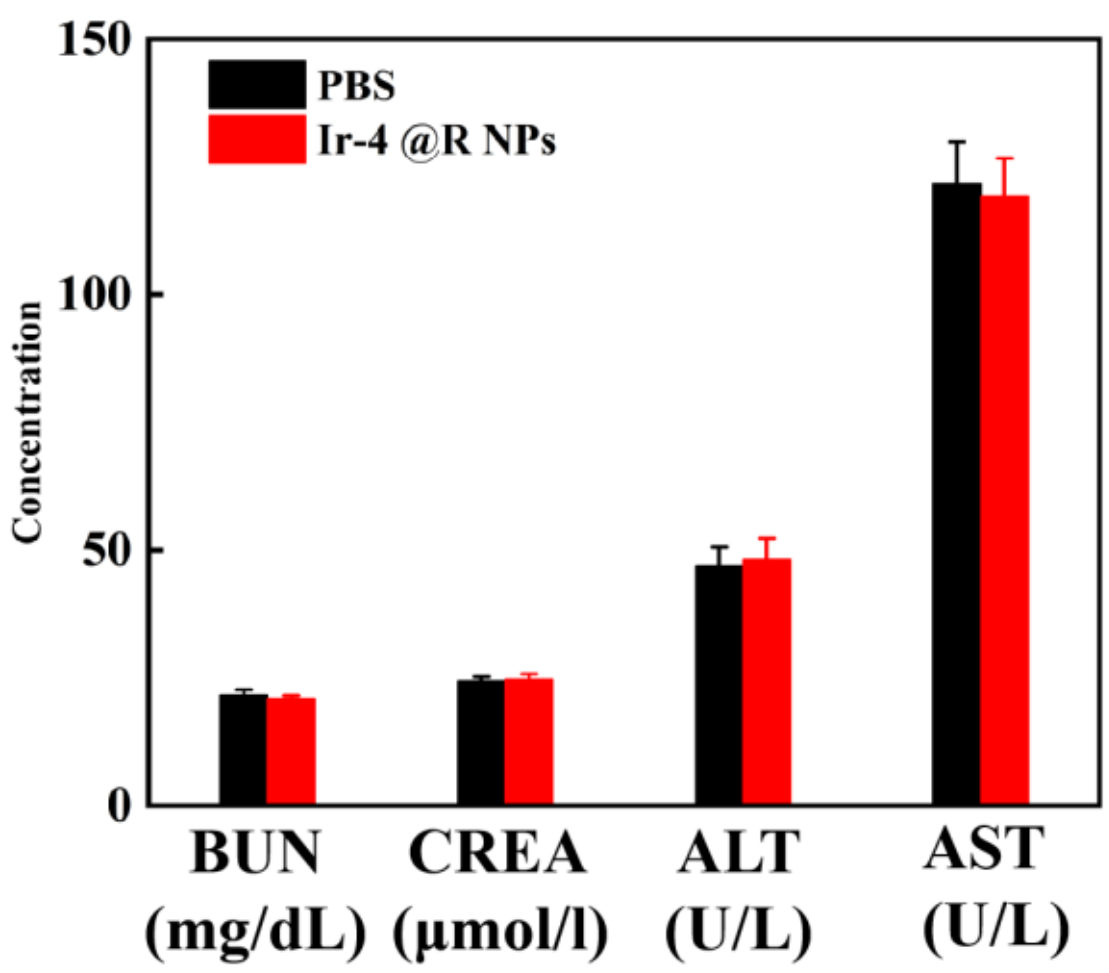

Figure S17. The hepatorenal indicator analysis of the mouse with different treatments. Data are represented as mean ± SD (n = 3). BUN is Blood Urea Nitrogen; CREA is Creatinine; ALT is Alanine Aminotransferase; AST is Aspartate Transferase.

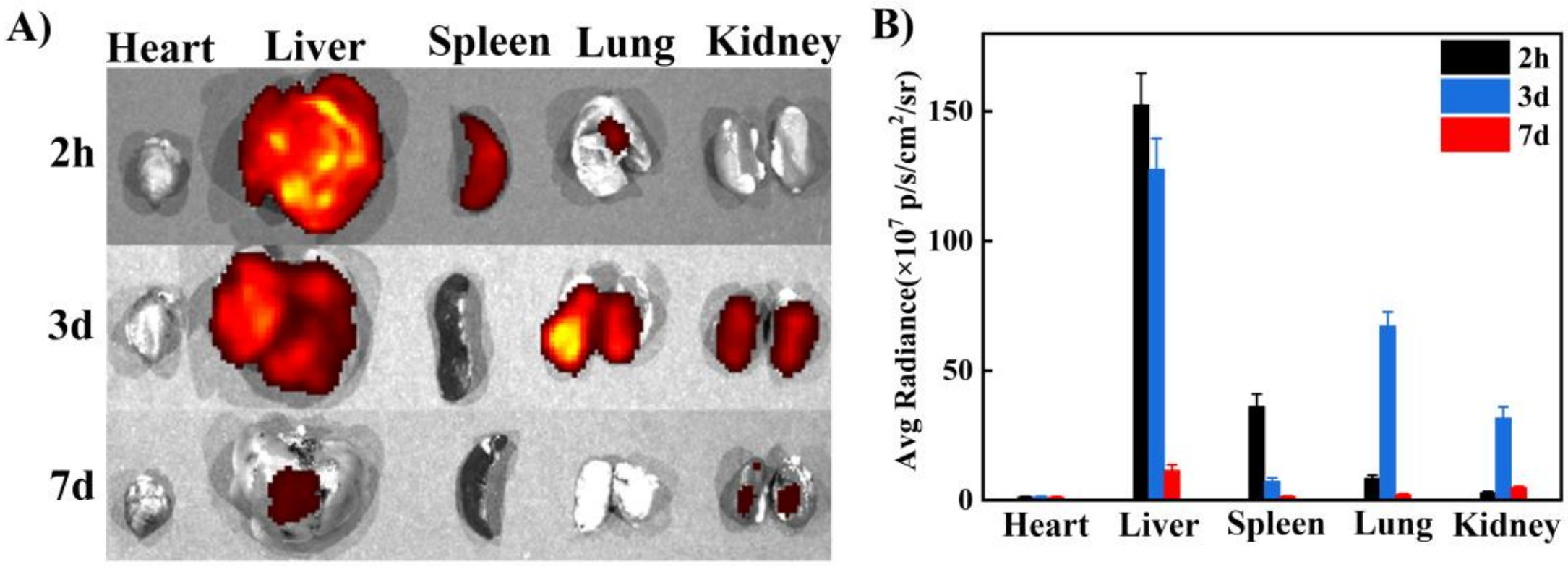

Figure S18. A) The fluorescence intensity of different mouse organs (left to right: heart, liver, spleen, lung and kidney) two hours, three days and seven days after injection of Ir-4@R NPs; B) Quantification of the fluorescence intensity of different organs. Data are presented as mean ± SD (n = 3 mice). The intensity of the brain's fluorescence was too weak to display.

[1] F. Neese, *WIREs Comput. Mol. Sci*. **2012**, *2*, 73–78.
[2] C. Adamo and V. J. Barone, *Chem. Phys*. **1999**, *110*, 6158–6170.
[3] S. Grimme, et al. *J. Chem. Phys*. **2010**, *132*, 154104.
[4] F. Weigend and R.Ahlrichs, *Phys. Chem. Chem. Phys*. **2005**, *7*, 3297–3305.
[5] K. Momma and F. Izumi, *J. Appl. Crystallogr.* **2011**, *44*, 1272–1276.
[6] A. Karton, et al. *J. Chem. Theory Comput*. **2017**, *13*, 1653–1665.
[7] E. Caldeweyher, et al. *J. Chem. Phys*. **2019**, *150*, 154122.
[8] T. Lu and F. Chen, *Computational and Theoretical Chemistry* **2021**, *1200*, 113249.
[9] G. Zhou, W.-Y. Wong, B. Yao, Z. Xie and L. Wang, *Angewandte Chemie International Edition* **2007**, *46*, 1149–1151.
